# Human skin specific long noncoding RNA *HOXC13-AS* regulates epidermal differentiation by interfering with Golgi-ER retrograde transport

**DOI:** 10.1038/s41418-023-01142-z

**Published:** 2023-03-03

**Authors:** Letian Zhang, Minna Piipponen, Zhuang Liu, Dongqing Li, Xiaowei Bian, Guanglin Niu, Jennifer Geara, Maria A. Toma, Pehr Sommar, Ning Xu Landén

**Affiliations:** 1 Dermatology and Venereology Division, Department of Medicine Solna, Center for Molecular Medicine, Karolinska Institutet, 17176 Stockholm, Sweden; 2 Key Laboratory of Basic and Translational Research on Immune-Mediated Skin Diseases, Chinese Academy of Medical Sciences, Jiangsu Key Laboratory of Molecular Biology for Skin Diseases and STIs, Institute of Dermatology, Chinese Academy of Medical Sciences and Peking Union Medical College, Nanjing, China; 3 Department of Plastic and Reconstructive Surgery, Karolinska University Hospital, Stockholm, Sweden; 4 Ming Wai Lau Centre for Reparative Medicine, Stockholm Node, Karolinska Institutet, 17176 Stockholm, Sweden

**Keywords:** Epigenetics, Epigenetics, RNA

## Abstract

After a skin injury, keratinocytes switch from a state of homeostasis to one of regeneration leading to the reconstruction of the epidermal barrier. The regulatory mechanism of gene expression underpinning this key switch during human skin wound healing is enigmatic. Long noncoding RNAs (lncRNAs) constitute a new horizon in the understanding of the regulatory programs encoded in the mammalian genome. By comparing the transcriptome of an acute human wound and skin from the same donor as well as keratinocytes isolated from these paired tissue samples, we generated a list of lncRNAs showing changed expression in keratinocytes during wound repair. Our study focused on *HOXC13-AS*, a recently evolved human lncRNA specifically expressed in epidermal keratinocytes, and we found that its expression was temporally downregulated during wound healing. In line with its enrichment in suprabasal keratinocytes, *HOXC13-AS* was found to be increasingly expressed during keratinocyte differentiation, but its expression was reduced by EGFR signaling. After *HOXC13-AS* knockdown or overexpression in human primary keratinocytes undergoing differentiation induced by cell suspension or calcium treatment and in organotypic epidermis, we found that *HOXC13-AS* promoted keratinocyte differentiation. Moreover, RNA pull-down assays followed by mass spectrometry and RNA immunoprecipitation analysis revealed that mechanistically *HOXC13-AS* sequestered the coat complex subunit alpha (COPA) protein and interfered with Golgi-to-endoplasmic reticulum (ER) molecular transport, resulting in ER stress and enhanced keratinocyte differentiation. In summary, we identified *HOXC13-AS* as a crucial regulator of human epidermal differentiation.

## Introduction

The epidermis is the outermost and stratified epithelium layer, and it protects the human body from external stimuli and prevents dehydration [1]. Keratinocytes constitute ~90% of all epidermal cells and undergo terminal differentiation to form the basal, spinous, granular, and cornified layers of the epidermis. Keratinocyte differentiation has been characterized by a dynamically changed gene expression program, e.g., early differentiated keratinocytes express KRT1, KRT10, and IVL, and then, LOR and FLG levels increase at later differentiation stages [2, 3]. Well-balanced keratinocyte proliferation and differentiation are essential for maintaining epidermal homeostasis, which is disrupted by skin injury, and wound-edge keratinocytes swiftly switch their status to engage in regeneration [4]. The dynamic gene expression and related regulatory mechanisms underpinning the switch between keratinocytes in the epidermal homeostasis state and the regeneration state are not fully understood; the mechanism is even more elusive in the human tissue environment during wound healing. Addressing fundamental questions about homeostasis-to-regeneration phenotype switching is required to understand the pathological mechanism underlying failed re-epithelization in chronic nonhealing wounds, which have led to major and increasing health and financial burdens worldwide [5, 6].

In addition to protein-coding genes, most of the human genome comprises a vast landscape of regulatory elements, including tens of thousands of long noncoding RNAs (lncRNAs). LncRNAs are transcripts longer than 200 nucleotides with no or limited sequences that can be translated [7, 8]. Increasing numbers of lncRNAs have been shown to regulate vital cellular processes via a large variety of molecular mechanisms and to play critical roles in health and disease [9]. Importantly, both the expression pattern of lncRNAs and their functions are more cell-type- and cell-state-specific than protein-coding genes, which endows lncRNAs with promising therapeutic and diagnostic potential [10–12]. In the skin, a few lncRNAs, including *ANCR*, *TINCR*, *LINC00941*, *uc.291*, and *PRANCR*, have been shown to regulate keratinocyte differentiation [13–17]. Moreover, three lncRNAs have been reported to function in keratinocytes during skin wound healing, i.e., *WAKMAR2* suppresses the inflammatory response, while *WAKMAR1*, *WAKMAR2*, and *TETILA* change cell mobility [18–20]. Although in its infancy, this field has produced evidence suggesting that lncRNAs are important regulators in epidermal homeostasis and regeneration, and further efforts to gain a more holistic and deeper understanding of these RNAs are warranted.

In this study, by tracing the in vivo transcriptomic changes in keratinocytes during human skin wound healing, we generated a list of lncRNAs that changed during the switch between the epidermal homeostasis state and the regeneration state. We focused on a human skin- and keratinocyte-specific lncRNA, *HOXC13-AS*, and revealed the crucial role it plays in regulating keratinocyte differentiation by interfering with retrograde protein transport from the Golgi to the endoplasmic reticulum (ER). The temporal downregulation of *HOXC13-AS* in wound-edge keratinocytes, likely due to high EGFR signaling during wound repair, and its restored expression during re-epithelialization reflect its physiological importance in the maintenance and reconstruction of the epidermal barrier.

## Results

### Downregulation of *HOXC13-AS* expression in human wound-edge keratinocytes

**Fig. 1:**
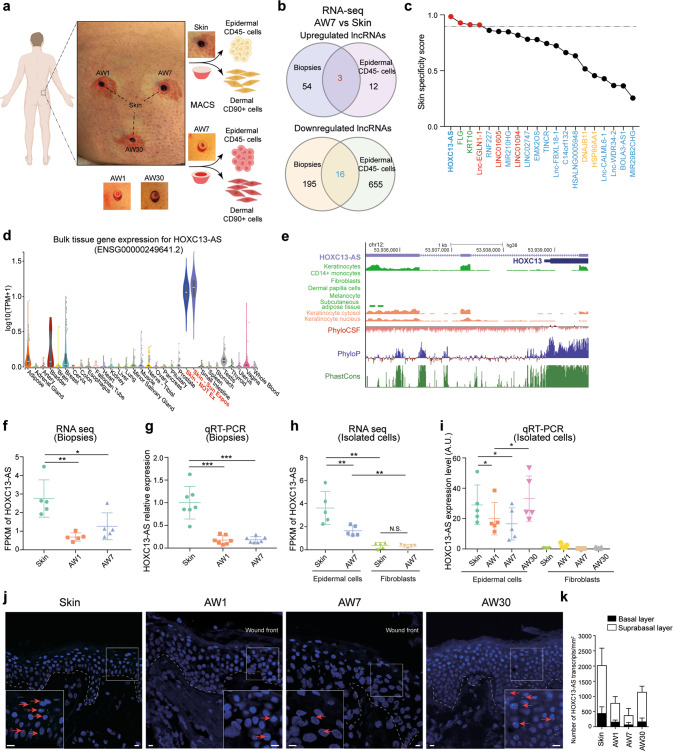
Downregulation of *HOXC13-AS* expression in human wound-edge keratinocytes. **a** Human in vivo wound model: full-thickness excisional wounds were created on the skin of healthy volunteers and wound-edge tissues were collected 1 (AW1), 7 (AW7), and 30 days later (AW30) from the same donor. Epidermal CD45^−^ cells (enriched with keratinocytes) and dermal CD90^+^ cells (fibroblasts) were isolated from the skin and AW7 biopsies by magnetic-activated cell sorting (MACS). RNA-sequencing was performed in both the tissues and the cells. **b** Venn diagram showing the differentially expressed lncRNAs in the CD45^−^ epidermal cells and the tissue biopsies of the AW7 compared to the skin (|Fold change| ≥ 2, *p* < 0.05). **c** Skin specificity scores of the differentially expressed lncRNAs surfaced in both the tissue and the epidermal cell RNA-seq analysis (upregulated in red, downregulated in blue), known skin-specific genes KRT10 and FLG (green), and broadly expressed genes DNAJB11 and HSP90AA1 (yellow). The dashed line indicates the skin specificity score of 0.9. **d** *HOXC13-AS* expression data across 31 normal human tissues retrieved from the GTEx database. **e** Genomic snapshot of *HOXC13-AS* generated in GENECODE V38. Data were retrieved from Encyclopedia of DNA Elements data hub, phylogenetic information-based codon substitution frequency (PhyloCSF), and conservation tracks (PhyloP and PhastCons). RNA-seq of *HOXC13-AS* in tissue biopsies (*n* = 5 donors) (**f**) and isolated cells (*n* = 5 donors) (**h**). Data are normalized as Fragments per kilobase of a transcript, per million mapped reads (FPKM). QRT-PCR analysis of *HOXC13-AS* in tissue biopsies (*n* = 7 donors) (**g**) and isolated cells (*n* = 5 donors) (**i**). Representative photographs (**j**) and quantification (**k**) of *HOXC13-AS* fluorescence in situ hybridization (FISH) in human skin and wounds (*n* = 2 donors). Cell nuclei were co-stained with DAPI (scale bar = 10 μm). *p < 0.05; **p < 0.01; ***p < 0.001 by Mann–Whitney test (**f**–**h**) and paired two-tailed Student’s *t* test (**i**). Data are presented as mean ± SD.

To characterize the role played by lncRNAs in human skin wound healing, we created wounds on the skin of healthy donors and collected the wound-edge tissues on day 1 (acute wound day 1, AW1), seven (AW7), and 30 (AW30) until the wounds closed (Fig. 1a). With ribosomal RNA-depleted long RNA-sequencing (RNA-seq) of these full-thickness tissue biopsy samples, we identified 57 upregulated and 211 downregulated lncRNAs on AW7 compared to the expression of these lncRNAs in matched skin from the five donors [|fold change| ≥ 2, False discovery rate (FDR) < 0.05, Fig. 1b and Supplementary Data 1]. To detect keratinocyte-related lncRNA expression changes, we conducted RNA-seq with epidermal CD45- cells and identified 15 upregulated and 671 downregulated lncRNAs in keratinocytes isolated from the AW7 wound edges compared to expression in the matched skin samples (|fold change| ≥ 2, FDR < 0.05, Fig. 1b and Supplementary Data 2). Interestingly, 3 upregulated and 16 downregulated lncRNAs were identified in both the tissue and epidermal cell RNA-seq analyses (Fig. 1b). We ranked these 19 lncRNAs on the basis of their skin specificity scores, which were calculated by the SPECS method [21] with the RNA-seq data obtained from 31 human normal tissues in the GTEx database [22]. HOXC13 antisense RNA (*HOXC13-AS*) was identified as a skin-specific lncRNA, with a score even higher than some known skin-specific genes, e.g., *FLG* and *KRT10* (Fig. 1c, d). The highly specific expression pattern of *HOXC13-AS* suggests its potentially unique function in the skin, which prompted us to take a closer look at this lncRNA.

*HOXC13-AS* is a divergent lncRNA transcribed from the opposite strand of the protein-coding gene *HOXC13* on human chromosome 12 [GRCh38/hg38, chr12:53,935,328–53,939,643] (Fig. 1e and Supplementary Fig. 1a). It is a recently evolved human lncRNA, as indicated by the low PhyloP and PhastCons scores, and no homolog in rodents has been identified. Moreover, a phylogenetic information-based codon substitution frequency analysis (PhyloCSF) suggested that *HOXC13-AS* lacks protein-coding potential, which was in agreement with a coding potential calculator CPC2 analysis [23] showing that the coding potential of *HOXC13-AS* was even lower than that of *HOTAIR* [24], a well-known lncRNA (Supplementary Fig. 1b).

Our RNA-seq analysis of human wounds revealed that *HOXC13-AS* expression was downregulated by AW1 and remained low through AW7 compared to that in the matching skin, and these findings were confirmed by real-time RT–PCR (qRT–PCR) analysis of tissue biopsy samples from seven other donors (Fig. 1f, g). Moreover, we performed RNA-seq and qRT–PCR with paired epidermal keratinocytes and dermal fibroblasts isolated from skin and wound samples on AW7 from ten healthy donors. We found that in human skin, *HOXC13-AS* was mainly expressed in keratinocytes but not in fibroblasts (Fig. 1h, i), which was in line with the public ENCODE data regarding *HOXC13-AS* expression in different cell types (Fig. 1e). Keratinocyte *HOXC13-AS* expression was transiently downregulated on AW1 and AW7 and then recovered to the level in paired skin on AW30, at which point the wounds had re-epithelized and showed epidermal stratification (Fig. 1i). Performing fluorescence in situ hybridization (FISH), we not only confirmed these findings but also localized *HOXC13-AS* mainly in the suprabasal layers of the epidermis (Fig. 1j, k and Supplementary Fig. 1e). Additionally, in published RNA-seq datasets of human wounds, we found reduced *HOXC13-AS* expression in diabetic foot ulcers compared to diabetic foot skin [25] and on day 2 and 5 in acute wounds compared to the expression in the skin [26] (Supplementary Fig. 1c, d). Consistent with its skin-specific expression (Fig. 1c, d), *HOXC13-AS* was not detected in human oral mucosal wounds [26] (Supplementary Fig. 1d).

In summary, we identified a human skin keratinocyte-specific lncRNA: *HOXC13-AS*. The expression of this lncRNA was reduced upon skin injury but was restored during re-epithelization, suggesting that *HOXC13-AS* may be involved in the maintenance and reconstruction of the epidermal barrier.

### Single-cell transcriptomic analysis of human skin revealed increased *HOXC13-AS* expression in granular keratinocytes

**Fig. 2:**
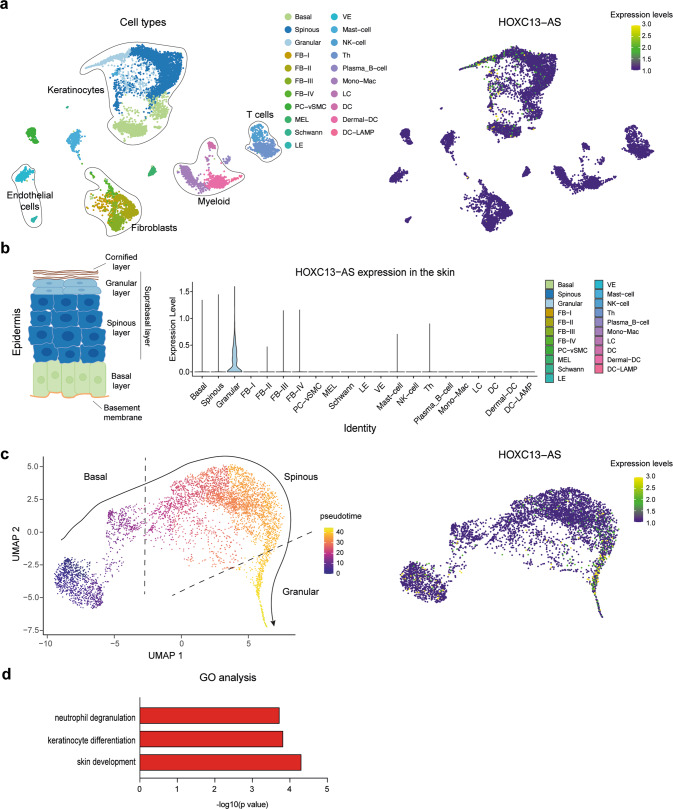
Granular keratinocytes are the major cells expressing *HOXC13-AS* in the skin. **a** UMAP representation of all cell types (left) and *HOXC13-AS* expressing cells (right) identified in human skin (*n* = 3 donors) by single-cell RNA-sequencing (scRNA-seq). **b** Violin plots of *HOXC13-AS* expression in different cell types in human skin. **c** Pseudotime trajectory of all the keratinocytes (i.e., basal, spinous, and granular keratinocytes) colored by pseudotime (left) and *HOXC13-AS* expression (right). **d** Gene Ontology (GO) analysis of the top 50 genes with expression positively correlated with *HOXC13-AS* in granular keratinocytes analyzed by scRNA-seq.

We performed a single-cell RNA-sequencing (scRNA-seq) analysis of human skin (*n* = 3) using 10X Chromium technology. Unsupervised clustering of 11800 cells with the Seurat package revealed 21 cell clusters, which included keratinocytes (basal, spinous, and granular keratinocytes), fibroblasts (FB-I–IV), melanocytes (MELs), Schwann cells, pericytes-vascular smooth muscle cells (PC-vSMCs), lymphatic and vascular endothelial cells (LE and VE), and immune cells [mast cells, NK cells, B cells, monocytes-macrophages (Monos-Macs), Langerhans cells (LC), and dendritic cells (DC)] (Fig. 2a). In line with the bulk RNA-seq data obtained with individual cell types (Fig. 1e, h), scRNA-seq demonstrated keratinocyte-specific *HOXC13-AS* expression in human skin (Fig. 2a). Interestingly, we found that *HOXC13-AS* was predominantly expressed in granular keratinocytes (Fig. 2b), which agreed with our FISH results showing higher *HOXC13-AS* expression in the suprabasal layers of the epidermis (Fig. 1j, k and Supplementary Fig. 1e). Additionally, a pseudotime analysis showed that all skin keratinocytes were ordered along a differentiation trajectory, which revealed that *HOXC13-AS* expression increased with keratinocyte differentiation (Fig. 2c). Notably, epidermal cell differentiation has been shown to be reduced during wound repair [27], which was confirmed here by analyzing late differentiation marker FLG expression in human acute wounds with immunofluorescence (IF) staining, as well as single-cell and spatial transcriptomic analysis, showing decreased FLG expression in wound-edge granular keratinocytes (Supplementary Fig. 1f–h). Therefore, *HOXC13-AS* expression changes with keratinocyte differentiation also during wound repair. Furthermore, to envisage the potential functional role of *HOXC13-AS*, we performed an expression correlation analysis between *HOXC13-AS* and all other genes expressed in the skin granular keratinocytes examined by the scRNA-seq, and thus, we identified 1935 positively correlated genes (*R* > 0, *p* < 0.05). Gene Ontology analysis (GO) unraveled that the 50 genes most correlated with *HOXC13-AS* were mainly involved in keratinocyte differentiation and immune response, suggesting that this lncRNA may play a role in these biological processes (Fig. 2d and Supplementary Data 3).

### *HOXC13-AS* expression is regulated in the opposite direction by keratinocyte growth and differentiation signaling

**Fig. 3:**
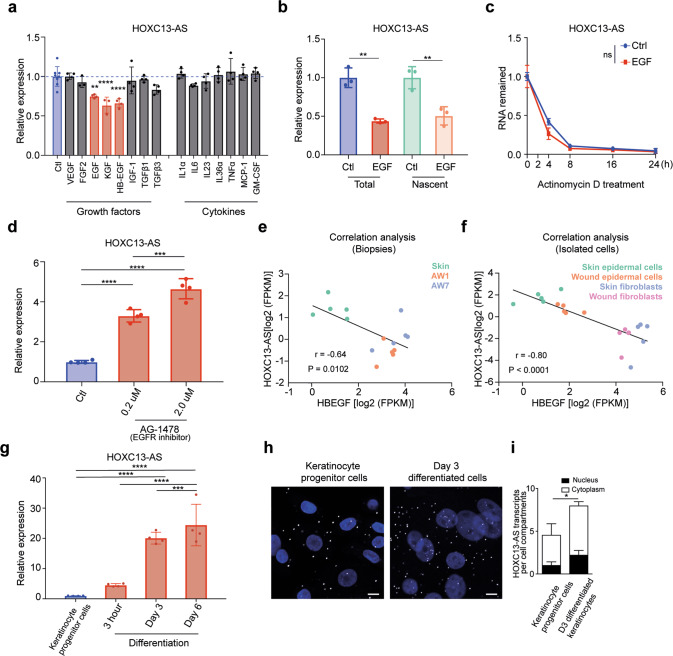
*HOXC13-AS* expression is regulated in the opposite direction by keratinocyte growth and differentiation signaling. **a** QRT-PCR analysis of *HOXC13-AS* expression in keratinocytes treated with wound-related cytokines and growth factors for 24 h (*n* = 4). **b** QRT-PCR analysis of total and nascent *HOXC13-AS* in keratinocytes treated with EGF for 8 h (*n* = 3). **c** QRT-PCR analysis of *HOXC13-AS* in keratinocytes treated with EGF and then actinomycin D for 0–24 h (*n* = 4). **d** QRT-PCR analysis of *HOXC13-AS* in keratinocytes treated with AG-1478 for 24 h (*n* = 4). Expression correlation of *HOXC13-AS* with *HBEGF* in the skin and wound tissues (**e**) and the isolated cells (**f**) analyzed by RNA-seq. **g** QRT-PCR analysis of *HOXC13-AS* in keratinocyte progenitor cells and calcium-induced differentiated keratinocytes (*n* = 4). Representative photographs (**h**) and quantification (**i**) of *HOXC13-AS* FISH in keratinocyte progenitor cells and differentiated keratinocytes treated with 1.5 mM calcium for 3 days (*n* = 3). Cell nuclei were co-stained with DAPI. Scale bar = 20 μm. ns not significant, *p < 0.05; **p < 0.01, ***p < 0.001 and ****p < 0.0001 by unpaired two-tailed Student’s *t* test (**a**, **b**, **d**, **g**, **i**), two-way ANOVA (**c**), or Pearson’s correlation test (**e** and **f**). Data are presented as mean ± SD.

We next explored the mechanism that modulates *HOXC13-AS* expression in epidermal homeostasis and regeneration. We treated human keratinocytes with a panel of growth factors (VEGF-A, FGF2, EGF, KGF, HB-EGF, IGF-1, TGF-β1, and TGF-β3) and cytokines (IL-1α, IL-6, IL-23, IL-36α, TNFα, MCP-1, and GM-CSF) known to be important to wound repair [28]. A qRT-PCR analysis revealed that *HOXC13-AS* expression was significantly downregulated by two members of the EGF family (HB-EGF and EGF) [28] and KGF (Fig. 3a and Supplementary Fig. 2a). Additionally, reduced *HOXC13-AS* expression was found in a public dataset (GSE156089) generated with an RNA-seq analysis of epidermal stem cells treated with EGF (Supplementary Fig. 2b). By labeling and purifying nascent RNA and then qRT-PCR detection of newly transcribed *HOXC13-AS*, we showed that EGF treatment reduced *HOXC13-AS* transcription in keratinocytes (Fig. 3b). Moreover, after blocking transcription with Actinomycin D, we found that EGF did not change *HOXC13-AS* stability (Fig. 3c). These data suggest that EGF may regulate *HOXC13-AS* expression at the transcriptional level. Furthermore, we showed that blocking EGF receptor (EGFR) signaling with the chemical inhibitor AG1478 [29] prominently enhanced *HOXC13-AS* expression in keratinocytes in a dose-dependent manner, which confirmed the inhibitory effect of growth factor signaling on *HOXC13-AS* expression in keratinocytes (Fig. 3d). Importantly, we found that the expression of HB-EGF and KGF was significantly upregulated during human skin wound repair and a significantly negative correlation between *HOXC13-AS* and *HBEGF* expression, as shown by the RNA-seq analysis of human wound tissues and isolated cells, suggesting that enhanced growth factor signaling may contribute to the downregulation of *HOXC13-AS* expression in human wounds in vivo (Fig. 3e, f and Supplementary Fig. 2c, d).

As the FISH and scRNA-seq analyses of human skin showed higher *HOXC13-AS* expression in more-differentiated keratinocytes (Figs. 1j, k and 2c) and because EGFR signaling inhibition has been reported to induce keratinocyte differentiation [30], we next examined whether differentiation enhanced *HOXC13-AS* expression in keratinocytes. Studying a calcium-induced keratinocyte differentiation model [31, 32], we found that *HOXC13-AS* expression gradually increased as cell differentiation progressed, as shown by both *HOXC13-AS* qRT–PCR and FISH analysis results (Fig. 3g–i and Supplementary Fig. 2e, f), which was also confirmed in a public transcriptomic analysis of differentiated keratinocytes at multiple time points (GSE59827) [33] (Supplementary Fig. 2g). Notably, differentiation also slightly reduced EGFR expression in keratinocytes, which may contribute to the differentiation-induced *HOXC13-AS* expression (Supplementary Fig. 2g, h).

Considering these data, we concluded that *HOXC13-AS* expression in keratinocytes was induced by cell differentiation but suppressed by growth factor signaling, which explains its increased expression in the suprabasal layers of the epidermis that contain more differentiated keratinocytes and its decreased expression during wound repair likely due to high growth signaling and decreased differentiation.

### *HOXC13-AS* promotes keratinocyte differentiation

**Fig. 4:**
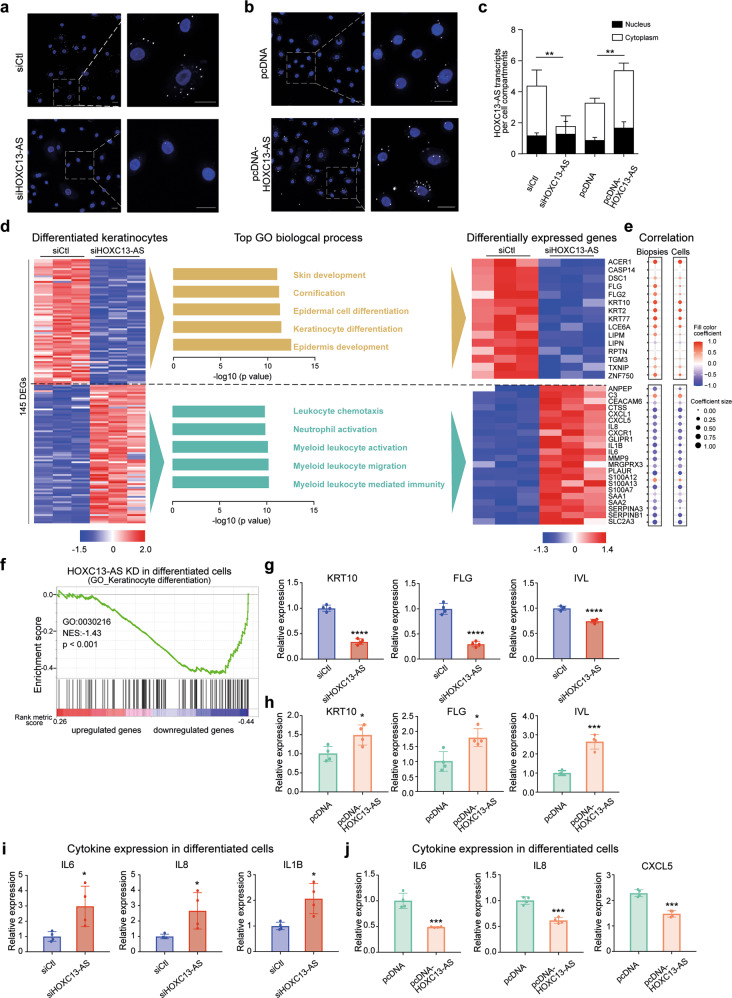
*HOXC13-AS* regulates keratinocyte differentiation and inflammatory response. Representative photographs (**a**, **b**) and quantification (**c**) of *HOXC13-AS* FISH in keratinocytes transfected with *HOXC13-AS* siRNA pool or nontargeting control (siCtl) (**a**), pcDNA-*HOXC13-AS* or empty vector (**b**) (*n* = 3). Cell nuclei were co-stained with DAPI. Scale bar = 20 μm. **d** Microarray analysis of differentiated keratinocytes transfected with *HOXC13-AS* siRNA pool or siCtl. Heatmap (left panel) illustrates the differentially expressed genes (DEGs, |Fold change| ≥ 2, FDR < 0.05). GO analysis of the DEGs is shown in the middle panel. The DEGs associated with the GO terms are shown in the heatmap (right panel). **e** Their expression correlation with *HOXC13-AS* in the skin and wound biopsies and isolated epidermal cells analyzed by RNA-seq. **f** GSEA evaluated enrichment for the keratinocyte differentiation-related genes (GO:0030216) in the microarray data. NES normalized enrichment score. QRT-PCR analysis of *KRT10*, *FLG* and *IVL* expression in differentiated keratinocytes transfected with *HOXC13-AS* siRNA pool (**g**) or pcDNA-*HOXC13-AS* (**h**) compared to respective controls (*n* = 4). QRT-PCR analysis of cytokine expression in differentiated keratinocytes transfected with *HOXC13-AS* siRNA pool (**i**) or pcDNA-*HOXC13-AS* (**j**) compared to respective controls (*n* = 4). *p < 0.05, **p < 0.01, ***p < 0.001, and ****p < 0.0001 by unpaired two-tailed Student’s *t* test (**c**, **g**–**j**), or Pearson’s correlation test (**e**). Data are presented as mean ± SD.

To characterize the potential functional role played by *HOXC13-AS*, we knocked down (KD) *HOXC13-AS* expression by transfecting human keratinocytes with *HOXC13-AS*-specific short interfering RNAs (siRNAs), and the loss of *HOXC13-AS* was confirmed by FISH (Fig. 4a, c) and qRT–PCR analyses (Supplementary Fig. 3a). In addition, we overexpressed (OE) *HOXC13-AS* in keratinocytes using a *HOXC13-AS* expression vector (pcDNA-*HOXC13-AS*), which significantly increased *HOXC13-AS* levels in the keratinocytes (Fig. 4b, c and Supplementary Fig. 3b). We showed that neither *HOXC13-AS* KD nor OE affected *HOXC13* expression in progenitor or differentiated keratinocytes (Supplementary Fig. 3c–e).

Next, we performed a microarray analysis with calcium-induced differentiated keratinocytes in which *HOXC13-AS* expression was knocked down, which led to the identification of 76 upregulated and 69 downregulated genes (|fold change| ≥ 2, *p* value < 0.05) (Fig. 4d). A GO analysis revealed that the expression of genes involved in keratinocyte differentiation (e.g., *KRT10*, *FLG*, *DSC1* [34], and *CASP14* [35]) was decreased, whereas the expression of immune response-related genes (e.g., *IL6, IL8*, and *IL1B*) was increased after *HOXC13-AS* was knocked down (Fig. 4d). Furthermore, we evaluated the expression of these *HOXC13-AS*-regulated genes in our RNA-seq datasets of human wound tissues and isolated epidermal cells (Fig. 1a). We found that differentiation- and inflammation-related genes were positively and negatively correlated with *HOXC13-AS* levels, respectively, supporting the in vivo relevance of our findings to *HOXC13-AS-*mediated gene regulation (Fig. 4e and Supplementary Fig. 3f–i). Additionally, a gene set enrichment analysis (GSEA) [36] confirmed that among the downregulated genes after *HOXC13-AS* was knocked down, keratinocyte differentiation-related genes (GO: 0030216) were significantly (*p* < 0.001) enriched (Fig. 4f). Furthermore, we confirmed the microarray findings by performing a qRT–PCR analysis of the keratinocyte differentiation markers (*KRT10, FLG*, and *IVL*) and the inflammatory genes (*IL6, IL8, IL1B, CXCL5*, *CXCL1*, and *MMP9)* in a calcium-induced keratinocyte differentiation model with *HOXC13-AS* OE or KD (Fig. 4g–j and Supplementary Fig. 3j). Both gain- and loss-of-function studies suggested that *HOXC13-AS* promoted keratinocyte differentiation while suppressing the cellular inflammatory response. Additionally, we showed that neither knocking down nor overexpressing *HOXC13-AS* affected human progenitor keratinocyte proliferation or migration (Supplementary Fig. 4a–c).

**Fig. 5:**
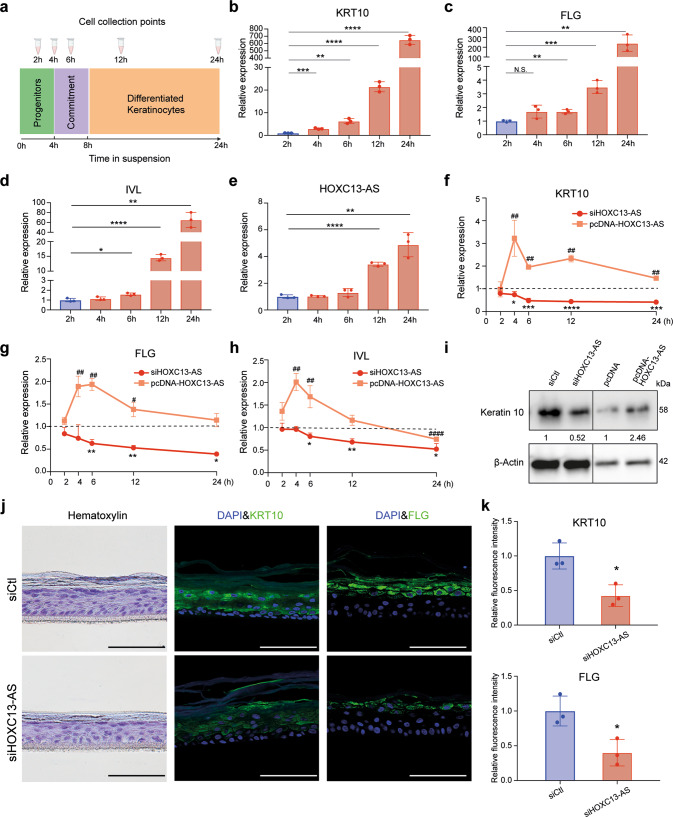
*HOXC13-AS* regulates keratinocyte differentiation in a suspension-induced differentiation model and organotypic human epidermal tissues. **a** Schematic representation of the experimental flow (left panel) and **b**–**e** qRT-PCR analysis of *KRT10*, *FLG*, *IVL*, and *HOXC13-AS* expression in the suspension-induced keratinocyte differentiation model (*n* = 3). QRT-PCR analysis of *KRT10* (**f**), *FLG* (**g**), and *IVL* (**h**) in keratinocytes transfected with *HOXC13-AS* siRNA pool, pcDNA-*HOXC13-AS* followed by suspension-induced differentiation. The results were normalized with the respective controls (*n* = 3). **i** Western blot of Keratin 10 in keratinocytes transfected with *HOXC13-AS* siRNA pool, pcDNA-*HOXC13-AS*, or respective controls after suspension induction for 24 h. **j** Representative photograph of hematoxylin and immunofluorescence staining of the organotypic epidermis with *HOXC13-AS* knockdown. Cell nuclei were co-stained with DAPI. Scale bar = 100 μm. **k** Quantification of fluorescence intensities of KRT10 and FLG in organotypic epidermis with *HOXC13-AS* knockdown (*n* = 3). * or ^#^*p* < 0.05; ** or ^##^*p* < 0.01, *** or ^###^*p* < 0.001 and **** or ^####^*p* < 0.0001 by unpaired two-tailed Student’s *t* test (**b**–**h**, **k**). Data are presented as mean ± SD.

Moreover, we utilized a suspension-induced keratinocyte differentiation model (Fig. 5a) [37, 38]. Human progenitor keratinocytes were cultured in a single-cell suspension from 2 to 24 h, which induced cell differentiation, as shown by the gradually increased expression of the differentiation markers *KRT10*, *FLG*, and *IVL* (Fig. 5b–d). Additionally, *HOXC13-AS* expression was enhanced through cell differentiation (Fig. 5e). After confirming the high efficiency of *HOXC13-AS* KD and OE in this model (Supplementary Fig. 4d), we showed that *HOXC13-AS* OE led to enhanced keratinocyte differentiation, whereas *HOXC13-AS* KD profoundly reduced differentiation, as evidenced by the changes in *KRT10*, *FLG*, and *IVL* at the mRNA (Fig. 5f–h) and protein levels (Fig. 5i).

To functionally characterize *HOXC13-AS* in a tissue environment, we generated organotypic human epidermal tissues [16] using progenitor keratinocytes with *HOXC13-AS* KD or OE. By performing IF staining, we showed that the abundance of the differentiation markers KRT10 and FLG, but not IVL, in suprabasal layer keratinocytes was significantly reduced after *HOXC13-AS* KD, whereas KRT10 and FLG expression were enhanced by *HOXC13-AS* OE, suggesting that modulation of *HOXC13-AS* changed keratinocyte differentiation in the organotypic epidermis (Fig. 5j, k and Supplementary Fig. 5a, b). Notably, in these organotypic tissues, we did not detect apoptotic cells through IF of caspase 3 (Supplementary Fig. 5c). The number of proliferating keratinocytes, which were localized to the basal layer of the epidermis, as indicated by Ki67 IF staining, was not changed by *HOXC13-AS* KD (Supplementary Fig. 5d).

Collectively, these data obtained with multiple physiologically relevant keratinocyte differentiation models demonstrated a prominent role for *HOXC13-AS* in promoting keratinocyte differentiation and showed that *HOXC13-AS* upregulation is required for maintaining the epidermal barrier.

### *HOXC13-AS* interacts with COPA protein

**Fig. 6:**
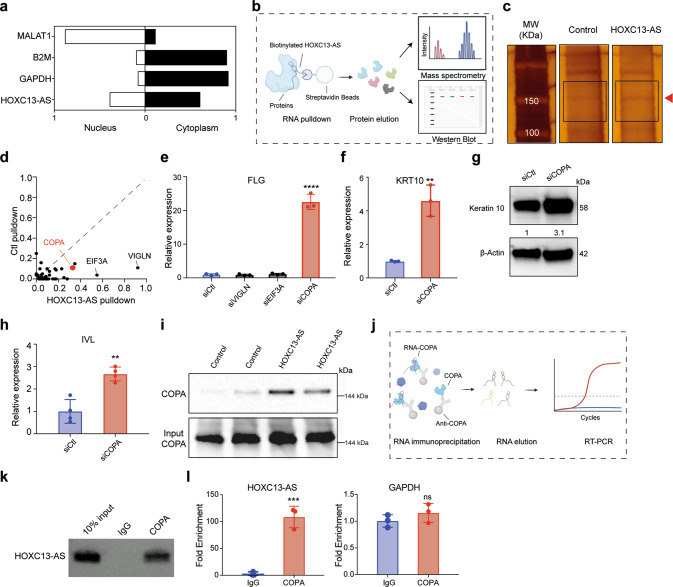
*HOXC13-AS* interacts with COPA protein to regulate keratinocyte differentiation. **a** QRT-PCR analysis of *HOXC13-AS*, *GAPDH*, *B2M*, and *MALAT1* in the nuclear or cytoplasmic fractions of keratinocytes. **b** Schematic representation of the RNA pulldown experiment. **c** Silver staining of proteins bound to *HOXC13-AS* or the control Poly(A)_25_ RNA. The red arrow indicates the differential band, and the rectangles specify the gel regions sent for mass spectrometry analysis (MS). **d** *HOXC13-AS* bound proteins identified by MS. **e** QRT-PCR analysis of *FLG* expression in differentiated keratinocytes with HDLBP, EIF3A, or COPA expression silencing (*n* = 3). QRT-PCR (**f**) and western blot (**g**) of Keratin 10 expression in differentiated keratinocytes with COPA knockdown (*n* = 3). **h** QRT-PCR of *IVL* expression in differentiated keratinocytes with COPA knockdown (*n* = 3). **i** Western blot of COPA bound to *HOXC13-AS* or control RNA. Same amounts of protein lysates were used as the input. **j** Schematic overview of the RNA immunoprecipitation assays (RIP). **k** Agarose gel electrophoresis of *HOXC13-AS* RT–PCR products with the RNA retrieved from RIP. **l** QRT-PCR analysis of *HOXC13-AS* and *GAPDH* retrieved from RIP using COPA antibody or IgG (*n* = 3). ns not significant, **p < 0.01, ***p < 0.001, and ****p < 0.0001 by unpaired two-tailed Student’s *t* test (**e**, **f**, **h**, and **l**). Data are presented as mean ± SD.

We next investigated the molecular mechanisms mediating the pro-differentiation function of *HOXC13-AS* in keratinocytes. In this analysis, subcellular localization is usually an indicator of the modes of action of lncRNAs. By performing cell fractionation assays, we showed a greater abundance of *HOXC13-AS* in the cytoplasm than in the nucleus of keratinocytes (Fig. 6a), which was consistent with our FISH analysis of human skin, wound tissues, and keratinocytes (Figs. 1j, 3h, i and 4a, b and Supplementary Fig. 1e), as well as the public ENCODE data (Fig. 1e). The cytoplasmic localization of *HOXC13-AS* indicated that it might act via an in trans mode [9, 39].

We surveyed the protein interactome of *HOXC13-AS* by performing an RNA pull-down experiment. After incubating keratinocyte protein lysates, biotinylated *HOXC13-AS* together with its binding proteins were purified in a process based on streptavidin beads [40] (Fig. 6b). Running the purified proteins in a gel followed by silver staining, we observed that a band representing ~150 kDa was more intense in the *HOXC13-AS* pull-down fraction than in the control poly(A)_25_ RNA pull-down fraction (Fig. 6c). We excised the regions of this differential band and analyzed the extracted protein by mass spectrometry (MS). High-density lipoprotein binding protein (VIGLN), eukaryotic translation initiation factor 3 subunit A (EIF3A), and coatomer subunit alpha (COPA) were identified as the proteins most enriched in the *HOXC13-AS* pull-down product fraction (Fig. 6d and Supplementary Data 4). As *HOXC13-AS* promotes keratinocyte differentiation, we next examined whether any of the identified proteins were involved in the same biological processes. By silencing their expression in keratinocytes with gene-specific siRNAs, we found that only knocking down *COPA* expression significantly changed *FLG* expression in differentiated keratinocytes (Fig. 6e). Furthermore, our qRT–PCR and western blotting analyses revealed that COPA silencing induced *KRT10* and *IVL* expression (Fig. 6f–h). Besides the calcium-induced differentiation model, we also confirmed that COPA silencing increased *KRT10*, *FLG, and IVL* expression in the suspension-induced keratinocyte differentiation model (Supplementary Fig. 6a). Therefore, our study focused on the COPA protein. We first confirmed the specific pull-down of the COPA protein by *HOXC13-AS* by western blotting with a COPA-specific antibody (Fig. 6i). Next, we performed RNA immunoprecipitation (RIP) (Fig. 6j), which showed that *HOXC13-AS*, but not *GAPDH*, was pulled down by an anti-COPA antibody but not by IgG (Fig. 6k, l and Supplementary Fig. 6b). These two-way ribonucleoprotein complex pull-down experiments provided strong evidence of the binding between *HOX1C3-AS* and COPA. Notably, silencing *HOXC13-AS* did not change COPA protein or mRNA levels in keratinocytes (Supplementary Fig. 6c, d), which was in line with our observation that COPA was evenly expressed across different epidermal layers in human skin (Supplementary Fig. 6e, f).

In summary, our data suggested that COPA negatively regulates keratinocyte differentiation and its binding with *HOXC13-AS* may serve as a key link in the mechanisms controlling epidermal differentiation.

### *HOXC13-AS* interferes with Golgi-ER retrograde transport causing ER stress

**Fig. 7:**
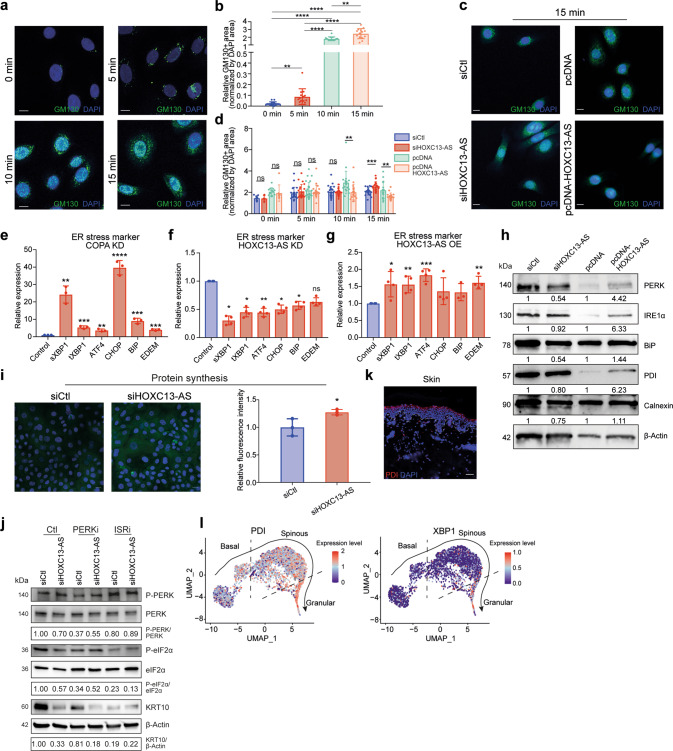
*HOXC13-AS* interferes with Golgi-ER retrograde transport causing ER stress. Representative photograph (**a**) and quantification (**b**) of immunofluorescence staining of GM130 in keratinocytes treated with Brefeldin A for 0–15 min. Cell nuclei were co-stained with DAPI. (Scale bar = 10 μm, *n* = 14–15 cells). Representative photograph (**c**) and quantification (**d**) of immunofluorescence staining of GM130 in keratinocytes transfected with *HOXC13-AS* siRNA pool, pcDNA-*HOXC13-AS* or respective controls, and treated with Brefeldin A. Cell nuclei were co-stained with DAPI (scale bar = 10 μm, *n* = 8–13 cells at 0 min and *n* = 18–22 cells at 5, 10, and 15 min). QRT-PCR analysis of ER stress markers in differentiated keratinocytes with COPA knockdown (KD, *n* = 3) (**e**), *HOXC13-AS* KD (*n* = 4) (**f**), or *HOXC13-AS* overexpression (OE, *n* = 4) (**g**). **h** Western blot of ER stress markers in keratinocytes transfected with *HOXC13-AS* siRNA pool, pcDNA-*HOXC13-AS* or respective controls after suspension induction for 24 h. **i** Protein synthesis assay in differentiated keratinocytes with *HOXC13-AS* KD (*n* = 3). **j** Western blot in differentiated keratinocytes with *HOXC13-AS* KD and treated with PERK inhibitors (PERKi) or inhibitors of the integrated stress response (ISRi). **k** Immunofluorescence staining of PDI in human skin. Cell nuclei were co-stained with DAPI. Scale bar = 50 μm. **l** *PDI* and *XBP1* expression in pseudotime trajectory of keratinocytes in human skin analyzed by single-cell RNA-seq. ns not significant, *p < 0.05, **p < 0.01, ***p < 0.001, and ****p < 0.0001 by unpaired two-tailed Student’s *t* test (**b**, **d**–**g**, and **i**). Data are presented as mean ± SD.

The COPA protein makes up a part of coatomer protein complex I (COPI), which is required for the retrograde transport of cargo proteins from the Golgi to the endoplasmic reticulum (ER) and the movement of vesicles within the Golgi [41]. As *HOXC13-AS* binds *to* COPA protein, we next evaluated whether *HOXC13-AS* affected the COPI-mediated retrograde transport in keratinocytes. To this end, we utilized brefeldin A (BFA), a fungal metabolite that blocks ER-to-Golgi transport [42], and assessed the extent of the redistribution of the Golgi marker GM130 from the Golgi to the ER [43]. Consistent with previous studies [44], GM130 IF showed that BFA treatment quickly induced Golgi-ER retrograde transport in keratinocytes (Fig. 7a, b). Interestingly, we found that *HOXC13-AS* KD enhanced and *HOXC13-AS* OE reduced GM130 retrograde transport (Fig. 7c, d and Supplementary Fig. 7a–c).

COPA mutations have been shown to cause ER stress that triggers the unfolded protein response (UPR) [45], which is an adaptive process for maintaining cell viability [46]. In brief, accumulation of unfolded/misfolded proteins in the ER lumen causes the ER-resident chaperone GRP78/BIP to dissociate from ER sensor proteins, i.e., protein kinase RNA-like ER kinase (PERK), inositol-requiring protein 1α (IRE1α), and activating transcription factor (ATF) 6. Activated PERK phosphorylates eukaryotic initiation factor (eIF) 2α, inhibiting global protein synthesis but increasing the translation of select mRNAs required for stress alleviation. Consistent with the previous findings [45], we showed that COPA silencing induced the expression of ER stress genes, such as *ATF4*, total XBP1 (*tXBP1*), spliced XBP1 (*sXBP1*) [47–49], C/EBP-homologous protein (*CHOP*), binding immunoglobulin protein (*BIP*) [50], and ER degradation enhancing alpha-mannosidase-like protein 1 (*EDEM1*) [51], in differentiated keratinocytes (Fig. 7e). Moreover, we found that *HOXC13-AS* KD reduced ER stress gene expression in calcium-induced differentiated keratinocytes, whereas *HOXC13-AS* OE increased ER stress gene expression in these cells (Fig. 7f, g). Similarly, a western blot analysis of a panel of well-established ER stress indicators, including PERK, IRE1α, and BIP, protein disulfide isomerase (PDI), and calnexin [46, 52, 53], revealed that *HOXC13-AS* KD inhibited and *HOXC13-AS* OE promoted the accumulation of these ER stress proteins in suspension-induced differentiated keratinocytes (Fig. 7h). Also, in organotypic human epidermal tissues, *HOXC13-AS* OE enhanced the expression of ER stress marker PDI, as shown by IF staining (Supplementary Fig. 5b). Furthermore, we showed that silencing *HOXC13-AS* increased general protein synthesis in differentiated keratinocytes induced by calcium, likely due to the alleviated ER stress (Fig. 7i). In line with this, western blot analysis unraveled that *HOXC13-AS* KD decreased the phosphorylation of PERK and eIF2α (Fig. 7j). Interestingly, treating cells with PERK or eIF2α inhibitors reduced KRT10 expression, which confirmed the role of UPR in keratinocyte differentiation [54] (Fig. 7j). However, only blocking eIF2α, but not PERK, revoked the impact of HOXC13-AS depletion on KRT10 expression, suggesting that PERK activation is not essential for the pro-differentiation effect of *HOXC13-AS* and other integrated stress response pathways that converge on eIF2α phosphorylation may be also involved [55] (Fig. 7j).

**Fig. 8:**
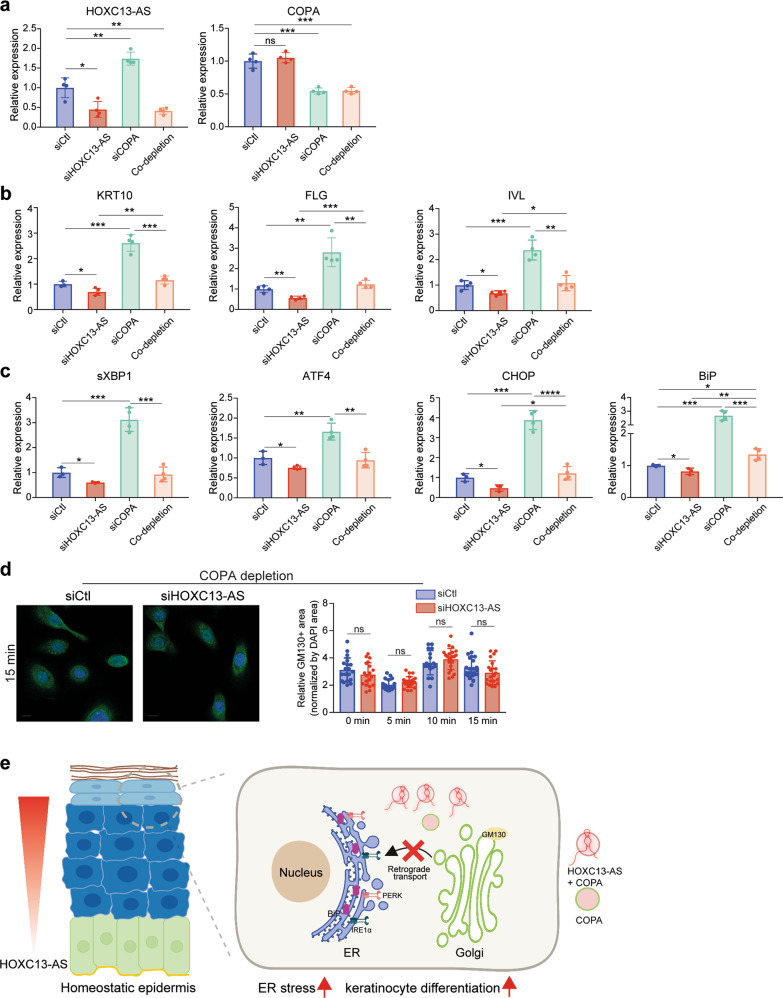
COPA is important in mediating the biological functions of *HOXC13-AS*. QRT-PCR analysis of *HOXC13-AS* and *COPA* (**a**), differentiation markers (**b**), or ER stress markers (**c**) in differentiated keratinocytes with individual *HOXC13-AS* or *COPA* KD, or co-depletion of *HOXC13-AS* and *COPA* (*n* = 3–4). **d** Representative photograph and quantification of immunofluorescence staining of GM130 in keratinocytes with *COPA* and *HOXC13-AS* KD, and then treated with Brefeldin A (*n* = 17–25). Cell nuclei were co-stained with DAPI. Scale bar = 10 μm. **e** A proposed model of *HOXC13-AS*/COPA-mediated regulation of keratinocyte differentiation. ns not significant, *p < 0.05, **p < 0.01, ***p < 0.001, and ****p < 0.0001 by unpaired two-tailed Student’s *t* test (**a**–**d**). Data are presented as mean ± SD.

Physiological ER stress has been known to play important roles in keratinocyte differentiation [56]. In line with previous studies [30, 57–59], we showed that the expression of ER stress markers *PDI* and *XBP1* was most enriched in the granular layer keratinocytes of human skin, suggesting that UPR is activated during epidermal differentiation (Fig. 7k, l). Additionally, pharmacological ER stressors, e.g., tunicamycin, have been shown to stimulate the expression of differentiation-related genes [30, 59]. Here, we found that tunicamycin treatment also elevated *HOXC13-AS* expression in keratinocytes (Supplementary Fig. 7d). Furthermore, we showed that *HOXC13-AS* level was enhanced by COPA silencing that promoted ER stress (Supplementary Fig. 7e). Importantly, we found that co-silencing *COPA* with *HOXC13-AS* rescued the repressive effects of *HOXC13-AS* KD on keratinocyte differentiation, ER stress, and GM130 retrograde transport, confirming the essential role of COPA in mediating *HOXC13-AS*’s biological functions (Fig. 8a–d). This constellation of findings suggests that by trapping COPA proteins, *HOXC13-AS* hampers Golgi–ER retrograde transport and leads to ER stress, thus promoting keratinocyte differentiation (Fig. 8e).

## Discussion

The search for gene expression regulatory mechanisms driving keratinocyte state switching between homeostasis and regeneration led to our identification of *HOXC13-AS*, a recently evolved, nonconserved human lncRNA. The epidermal-specific expression of *HOXC13-AS* raised the possibility that it may have evolved to regulate epidermis-related functions. As shown in our study, *HOXC13-AS* plays a crucial role in promoting keratinocyte differentiation, leading to the establishment of an effective epidermal barrier. To the best of our knowledge, this is the first report showing the physiological role played by *HOXC13-AS*. This lncRNA has been previously identified as a cancer biomarker in cervical cancer [60], breast cancer [61], glioma [62], hepatocellular carcinoma [63], cholangiocarcinoma [64], and head and neck squamous cell carcinoma [65–67], which emphasizes the absence of *HOXC13-AS* expression in most normal tissues except the skin. Additionally, *HOXC13-AS* reportedly functions as an oncogene promoting proliferation and the epithelial-mesenchymal transition of cancer cells [65]. However, we did not observe a significant effect on normal keratinocyte migration or proliferation (Supplementary Fig. 4a–c). Importantly, our study revealed the highly specific expression and functional pattern of *HOXC13-AS* in human skin, which is a crucial feature endowing lncRNAs with promising therapeutic and diagnostic potential [10–12].

Our study adds *HOXC13-AS* to the short list of lncRNAs known to modulate epidermal cell differentiation [13–16], which includes both negative (*ANCR* and *LINC00941*) and positive regulators (*TINCR*, *uc.291*, and *PRANCR)* [13–17]. Among these lncRNAs, only *TINCR*, similar to *HOXC13-AS*, was downregulated in human wound tissues and wound-edge keratinocytes compared to the skin in our RNA-seq analysis (Fig. 1b, c). Prior studies have shown that *TINCR* directly binds to the STAU1 protein, thus stabilizing differentiation-related mRNAs [14]. In addition to acting as a lncRNA, *TINCR* encodes a protein named TINCR-encoded ubiquitin-like protein (TUBL), which promotes keratinocyte proliferation and wound repair [68, 69]. Further study to determine whether TINCR may play a role in keratinocyte homeostasis-to-regeneration state transition is warranted, and if TINCR is found to be involved, the determination of whether it functions as a lncRNA or TUBL in this process will be needed.

We discovered that *HOXC13-AS* acts via a unique mechanism, i.e., sequestration of COPA, a protein required for retrograde Golgi-to-ER transport to recycle the ER-derived transport machinery and resident proteins [70, 71]. Mutant COPA has been previously shown to impair the assembly of proteins targeted for transport and to lead to ER stress and UPR activation in hereditary autoimmune-mediated lung disease and arthritis [41, 45]. Previous studies have demonstrated that physiological ER stress is required to modulate keratinocyte differentiation [56]. Our study not only confirmed the importance of COPA in maintaining ER homeostasis but also uncovered its inhibitory role on keratinocyte differentiation. We revealed a novel regulatory mechanism for COPA-mediated Golgi-to-ER retrograde transport, i.e., the lncRNA *HOXC13-AS* traps COPA protein, thus hindering COPA-mediated cargo assembly, which results in ER stress and promotes keratinocyte differentiation (Fig. 8e). In contrast to the physiological levels of ER stress that are required for modulation of keratinocyte differentiation, persistent or excessive levels of ER stress lead to cell death and apoptosis, which has been detected in skin diseases with aberrant epidermal differentiation, such as Darier’s disease [72]. It is tempting to explore whether *HOXC13-AS* may play a pathological role or even serve as a therapeutic target in human diseases with chronic ER stress.

During human skin wound healing, the expression of *HOXC13-AS* in wound-edge keratinocytes was transiently downregulated, likely due to high EGFR signaling in the wound environment. It has been previously shown that sustained activation of EGFR signaling suppressed keratinocyte differentiation, whereas its blockade induced differentiation through the activation of Notch signaling [29]. In this study, we added an additional mechanistic link between EGFR signaling and epidermal differentiation, i.e., EGFR signaling inhibited the expression of *HOXC13-AS*, a crucial positive regulator of keratinocyte differentiation. In addition to its differentiation-promoting function, *HOXC13-AS* suppressed the innate immune response in keratinocytes; thus, its rapid downregulation upon skin injury may facilitate the initiation of the inflammatory stage of wound repair (Supplementary Fig. 3k). Moreover, in homeostatic skin, the enrichment of *HOXC13-AS* in differentiated keratinocytes, which comprise the outermost layers of the epidermis, may contribute to the maintenance of immune tolerance of the skin barrier, which is constantly exposed to the harsh external environment.

Taken together, the data obtained through our study shows that a human-specific lncRNA, *HOXC13-AS*, is a crucial regulator of epidermal differentiation and that it functions by sequestrating the COPA protein and interfering with Golgi-to-ER transport. These findings contribute to understanding the molecular mechanisms required to maintain and regenerate the epidermal barrier. The specific expression in human skin and the critical function in regulating ER stress make *HOXC13-AS* a potential therapeutic target for a range of cutaneous diseases characterized by chronic ER stress.

## Supplementary information

aj-checklist

Supplementary Material

Uncropped WB

Dataset 1

Dataset 2

Dataset 3

Dataset 4

## Data Availability

Raw data of long RNA-sequencing and microarray performed for this study have been deposited to NCBI’s Gene Expression Omnibus (GEO) database under the accession numbers GSE174661 and GSE206103, respectively.
