# Supplementary material for: Human skin specific long noncoding RNA HOXC13-AS regulates epidermal differentiation by interfering with Golgi-ER retrograde transport
Source: Cell Death Differ. 2023 Mar 3;30(5):1334–48. doi: 10.1038/s41418-023-01142-z (PMC10154349; doi:10.1038/s41418-023-01142-z)
Supplement: Supplementary file 2 — Supplementary Material [file 41418_2023_1142_MOESM2_ESM.pdf]

Supplementary Information

Human skin specific long noncoding RNA *HOXC13-AS* regulates epidermal differentiation by interfering with Golgi-ER retrograde transport

Letian Zhang[1], Minna Piipponen[1], Zhuang Liu[1], Dongqing Li[1,2], Xiaowei Bian[1], Guanglin Niu[1], Jennifer Geara[1], Maria A. Toma[1], Pehr Sommar[3] and Ning Xu Landén[1,4]*

[1]Dermatology and Venereology Division, Department of Medicine Solna, Center for Molecular Medicine, Karolinska Institutet, 17176 Stockholm, Sweden

[2]Institute of Dermatology, Chinese Academy of Medical Sciences and Peking Union Medical College, Nanjing, Jiangsu, China

[3]Department of Plastic and Reconstructive Surgery, Karolinska University Hospital, Stockholm, Sweden

[4]Ming Wai Lau Centre for Reparative Medicine, Stockholm Node, Karolinska Institute, 17177 Stockholm, Sweden

To whom correspondence should be addressed: email: ning.xu@ki.se

This PDF file includes:

Methods
Supplementary Figures 1–7
Supplementary Tables 1–4

METHODS

Human *in vivo* wound healing model

Healthy donors (Supplementary Table 1) were recruited, and the surgical procedures were conducted at Karolinska University Hospital (Stockholm, Sweden). Two or three full-thickness excisional wounds were made on the skin of each donor by using a 4 mm biopsy punch, and the excised skin was collected as baseline control (Skin). The wound-edge tissue was collected by using a 6 mm biopsy punch one (AW1), seven (AW7), and thirty days later (AW30) (Fig. 1a). Local lidocaine injection was used for anesthesia while sampling. Written informed consent was obtained from all the donors for collecting and using clinical samples. The study was approved by the Stockholm Regional Ethics Committee (Stockholm, Sweden) and conducted according to the Declaration of Helsinki's principles.

Cell culture and treatments

Human adult epidermal keratinocytes were cultured in EpiLife medium supplemented with 10% Human Keratinocyte Growth Supplement (HKGS) and 1% Penicillin and Streptomycin at 37℃ in 5% CO_2 (all from ThermoFisher Scientific, Waltham, MA). Calcium concentration was increased to 1.5 mM in the medium to induce cell differentiation. To study the mechanisms regulating *HOXC13-AS* expression, we treated keratinocytes with EGFR inhibitor AG-1478 (Millipore, Burlington, MA), cytokines and growth factors (Supplementary Table 2), or PBS as a control for 24 hours, and then *HOXC13-AS* expression was analyzed by qRT-PCR. To study if EGF influences *HOXC13-AS* transcription, Click-iT™ Nascent RNA Capture Kit (ThermoFisher Scientific) was used. Keratinocytes were treated with EGF for 8 hours and 5-ethynyl uridine of 0.5 mM for 1 hour, and then nascent RNAs were captured and purified according to the manufacturer's instructions. To study if EGF affects *HOXC13-AS* stability, we treated keratinocytes with EGF and actinomycin D (5 µg/ml) for multiple time points, and *HOXC13-AS* levels were examined by qRT-PCR. To study the biological function of *HOXC13-AS*, we transfected the third passage of keratinocytes at 60-70% confluence with a 10 nM siRNA pool targeting *HOXC13-AS* or negative control siRNAs (Supplementary Table 3) for 24 hours with Lipofectamine™ 3000 (ThermoFisher Scientific). To identify the function of *HOXC13-AS*-bound proteins, we transfected keratinocytes with 20 nM siRNAs targeting COPA, HDLBP, EIF3A, or negative control siRNAs for 24 hours (Supplementary Table 3).

Migration and proliferation assays

Human primary keratinocytes were plated in Essen ImageLock 96-well plates (Sartorius, Göttingen, Germany) at 15,000 cells per well for migration assay and in 12-well plates at 20,000 cells per well for cell growth assay. In the migration assay, the confluent cell layers were pretreated with Mitomycin C (sigma aldrich, St. Louis, MO) for two hours, then scratched by using Essen wound maker. The plates were placed into IncuCyte (Sartorius) and imaged every two hours. The photographs were analyzed by using the IncuCyte software (Sartorius). Cell proliferation was also assessed by 5-ethynyl-2'-deoxyuridine (EdU) incorporation assay using Click-iT™ EdU Alexa Fluor™ 647 Flow Cytometry Assay Kit (ThermoFisher Scientific) according to the manufacturer's instructions.

RNA extraction and qRT-PCR

Before RNA extraction, skin and wound biopsies were homogenized using TissueLyser LT (Qiagen, Hilden, Germany). Total RNA was extracted from human tissues and cells using the Trizol reagent (ThermoFisher Scientific). Reverse transcription was performed using the RevertAid First Strand cDNA Synthesis Kit (ThermoFisher Scientific). Gene expression was determined by TaqMan expression assays (ThermoFisher Scientific) or by SYBR Green expression assays (ThermoFisher Scientific) and normalized based on the values of the housekeeping gene *B2M* or *GAPDH*. Information for all the primers used in this study is listed in Supplementary Table 4.

Magnetic cell separation of CD45- epidermal cells

After being washed in PBS, the skin and wound tissues were incubated in dispase II (5 U/mL, ThermoFisher Scientific) at 4°C overnight, and the epidermis and the dermis were separated. The epidermis was cut into small pieces and digested in 0.025% Trypsin/EDTA Solution at 37°C for 15 minutes. CD45- and CD45+ cells were separated using CD45 Microbeads with MACS MS magnetic columns according to the manufacturer's instructions (Miltenyi Biotec, Bergisch Gladbach, Germany).

Single-cell RNA sequencing

Skin and wounds were incubated in dispase II (5 U/mL, ThermoFisher Scientific) at 4°C overnight, and the epidermis and the dermis were separated. Single dermal cells were obtained using a whole skin dissociation kit (Miltenyi Biotec). Single epidermal cells were collected by incubating in 0.025% Trypsin-

EDTA at 37°C for 15-20 minutes. After removing red blood cells, an equal number of epidermal and dermal cells were mixed. The dead cells were removed using the MACS Dead cell removal kit (Miltenyi Biotec). Single cells were loaded onto each channel of the Single Cell chip (10x Genomics, Pleasanton, CA) before droplet encapsulation on the Chromium Controller. Sequencing libraries were generated using the library construction kit (10x Genomics) following the manufacturer's protocol. Libraries were sequenced on Illumina NovaSeq 6000 S4 v1.5. Raw sequencing data were processed with the cellranger pipeline (10X Genomics, version 5.0.1) and mapped to the hg38 reference genome to generate matrices of gene counts by cell barcodes.

Gene-barcode counts matrices were analyzed with the Seurat R package (version 4.0.6)(1). Genes expressed in less than ten cells and cells with < 500 genes detected, < 1000 UMI counts, or > 20% mitochondrial gene mapped reads were filtered away from downstream analysis. The Seurat object of each sample was normalized and scaled with R package sctransform(2). PCA was performed on the top 4000 variable genes using RunPCA, and the first 40 principal components (PCs) were used in the function of RunHarmony from the Harmony package(3) to remove potential confounding factors among samples processed in different libraries. UMAP plots were generated using the RunUMAP function with the first 40 harmonies. The clusters were determined by using the FindNeighbors and FindClusters functions with a resolution of 0.8.

Bulk RNA-sequencing

Ribosomal RNAs (rRNA) were removed using the Epicentre Ribo-zero® rRNA Removal Kit (Epicentre, Madison, WI). For the whole biopsies, strand-specific total-transcriptome RNA sequencing libraries were prepared by incorporating dUTPs in the second-strand synthesis using NEB Next® Ultra^{TM} Directional RNA Library Prep Kit for Illumina® (NEB, Ipswich, MA). The isolated cell RNA-seq libraries were prepared using the Ovation® SoLo® RNA-Seq library preparation kit (Tecan, Männedorf, Switzerland). The libraries were sequenced on the Illumina Hiseq 4000 platform (Illumina, San Diego, CA).

Raw reads of RNA sequencing were first cleaned by using Trimmomatic v0.36 software(4). Cleaned reads were mapped to the human reference genome (GRCh38.p12) and the comprehensive gene annotation file (GENCODEv31) using STAR v2.7.1a(5). Gene expression was then quantified by calculating unique mapped fragments to exons using the Subread package(6). The differential

expression analysis was performed using DESeq2(7). The differentially expressed RNAs were defined as $P < 0.05$, and |Fold change| >= 2. Raw counts for each gene were normalized to fragments per kilobase of a transcript, per million mapped reads (FPKM)-like values.

Spatial transcriptomics

Spatial transcriptomics was performed using fresh skin and wounds following instructions of Visium Spatial platform of 10x Genomics. In brief, we firstly performed H&E staining to assess the morphology and quality of tissues. Permeabilization was performed followed by reverse transcription and second strand synthesis. cDNA Library preparation, clean up, and indexing was then conducted following the manufacturer's instructions. The pooled libraries are sequenced on NovaSeq6000 S4-200 (Illumina) generating ~300 M reads per section. Space Ranger 1.2 (10x Genomics) was used to process the raw ST data, using the GRCh38 human reference genome alignment and GENCODE v38 gene annotation, and visualized by BioTuring's BBrowser software.

***In situ* hybridization**

HOXC13-AS probe (Cat. No. 486901) was designed and synthesized by Advanced Cell Diagnostics (ACD, Silicon Valley, CA). Tissues and cells were prepared by following the manufacturer's instructions. After fixation and dehydration with 50%, 70%, and 100% ethanol, the slides were incubated with Protease IV (ACD) at room temperature for 30 minutes. Then the slides were incubated with *HOXC13-AS* probes for two hours at 40 °C in HybEZ™ II Hybridization System using RNAscope® Multiplex Fluorescent Reagent Kit v2 (ACD). The hybridization signals were amplified via sequential hybridization of amplifiers and label probes. *HOXC13-AS* signals were visualized on Zeiss LSM800 confocal microscope (Oberkochen, Germany) and analyzed with ImageJ software (National Institutes of Health).

Western blotting

Keratinocyte cell lysates were extracted using radioimmunoprecipitation assay (RIPA) buffer complemented with protease inhibitor and phosphatase inhibitor. The total protein was separated in TGX™ precast protein gels (Bio-Rad, Hercules, CA) and transferred to a nitrocellulose membrane. Blots were probed with antibodies for anti-KRT10 (ab76318, 1:1000 dilution, Abcam, Cambridge, United Kingdom), anti-eIF2α (ab242148, 1:1000 dilution, Abcam), anti-P-eIF2α (ab32157, 1:1000 dilution,

Abcam), anti-PERK (5683, 1:1000 dilution, CST, Danvers, MA), anti-P-PERK (bs-3330R, 1:1000 dilution, Bioss Antibodies, Woburn, MA), anti-COPA (sc-398099, Santa Cruz Biotechnology, Dallas, TX), or ER stress markers (9956, dilution following manufacturer's instructions, CST). Next, the blots were incubated with anti-mouse (P0447, 1:1000 dilution, DAKO, Denmark) or anti-rabbit (P0448, 1:2000 dilution, DAKO) HRP conjugated secondary antibodies. β-actin levels were used as an internal control in each sample (A3854, 1:20,000 dilution, Sigma-Aldrich, St. Louis, MO). Protein bands were quantified with ImageJ and Image lab (Bio-Rad).

Suspension-induced keratinocyte differentiation

Keratinocytes differentiate in suspension, as described previously(8). We suspended keratinocytes at a concentration of 10^5 cells/mL in the EpiLife medium containing 1.45% methylcellulose (Sigma-Aldrich) in 24-well plates at 37°C. At different time points after cell suspension, the methylcellulose was diluted with PBS and the cells were recovered by centrifugation.

Immunofluorescence staining

Cultured cells were fixed with 4% paraformaldehyde for 15 minutes at room temperature and permeabilized with 0.1% Triton X-100. The paraffin-embedded organotypic epidermis and human skin tissues were deparaffinized and rehydrated before staining. Cells and tissues were blocked by 5% bovine serum albumin (BSA) in PBS for 30 minutes at room temperature, followed by immunostaining with anti-Ki67 (9129, 1:400 dilution, CST), anti-GM130 (PA1-077, 1:500 dilution, ThermoFisher Scientific), anti-KRT10 (M7002, 1:200 dilution, DAKO), anti-PDI (3501, 1:100 dilution, CST), or anti-FLG antibodies (MA513440, 1:100 dilution, ThermoFisher Scientific) at 4℃ overnight. On the next day, the sections were washed three times with PBS and then incubated with donkey anti-mouse or anti-rabbit secondary antibody conjugated with Alexa Fluor™ 488 (A-21202 and A-21206, 1:1000 dilution, Invitrogen) in the dark at room temperature for 40 minutes. After being washed with PBS three times, the sections were mounted with the ProLong™ Diamond Antifade Mountant with DAPI (ThermoFisher Scientific). Samples were visualized by Zeiss LSM800 confocal microscopy and analyzed with ImageJ software.

Protein synthesis assay

To study if *HOXC13-AS* affects total protein synthesis during keratinocyte differentiation, we used Click-iT™ HPG Alexa Fluor™ 488 Protein Synthesis Assay Kit (ThermoFisher Scientific). Briefly, keratinocytes transfected with the *HOXC13-AS* siRNA pool were induced for differentiation for 24 hours, and then cells were incubated with Click-iT™ HPG (50 μM) for 30 min. Cell fixation, permeabilization, and Click-iT™ HPG were performed following manufacturer's instructions.

Gene expression microarray and analysis

Keratinocytes were transfected with either *HOXC13-AS* siRNA or control siRNA for 24 hours (n=3 per group) and transcriptomic profiling was performed using human Clariom™ S assays (ThermoFisher Scientific) at the core facility for Bioinformatics and Expression Analysis (BEA) at Karolinska Institute. In brief, total RNA was extracted using the miRNeasy Mini Kit (Qiagen), and RNA quality and quantity were determined using Agilent 2200 Tapestation with RNA ScreenTape (Agilent, Santa Clara, CA) and Nanodrop 1000 (ThermoFisher Scientific). 150 ng of total RNA was used to prepare cDNA following the GeneChip WT PLUS Reagent Kit labeling protocol (ThermoFisher Scientific). Standardized array processing procedures recommended by Affymetrix, including hybridization, fluidics processing, and scanning, were used. Genes showing at least 2-fold change and with a P value less than 0.05 were considered differentially expressed. Gene set enrichment analysis (GSEA) was performed using a public software from Broad Institute(9). MetaCore software (Thomson Reuters, Toronto, Canada) was used for Gene Ontology (GO) analysis. Heatmaps were generated with the Multiple Experiment Viewer software.

Organotypic epidermis culture

The organotypic epidermis was cultured as described previously(10). 1.55×10^5 human primary keratinocytes were resuspended in a CnT-PR medium (CELLnTEC, Bern, Switzerland) and placed onto 0.47 cm^2 inserts with 0.4 μm pore size (Nunc, Roskilde, Denmark). Six inserts were put into a 60 mm cell culture dish filled with the CnT-PR medium. When cells reached confluency, the medium inside and outside the insert was replaced with a CnT-PR-3D medium (CELLnTEC). Twenty-four hours later, the medium inside was removed, and the surface of the inserts remained dry for the rest of the cultivation. Medium outside was changed every two days. Inserts were collected on day 12 after airlifting and followed by fixation and embedding.

Cellular fractionation

Nuclear and cytoplasmic fractions were separated by PARIS kit following manufacturer's instructions (ThermoFisher Scientific). RNA was extracted from these fractions by using Trizol (ThermoFisher Scientific) and qRT-PCR was performed to analyze the *MALAT1*, *B2M*, *GAPDH,* and *HOXC13-AS* expression.

***HOXC13-AS* overexpression construct**

For the generation of the *HOXC13-AS* expression vector, the *HOXC13-AS* fragment in pEX-A258 was obtained from Eurofins Genomics (Ebersberg, Germany) and cloned into pcDNA3.1(+) vector (Invitrogen, Carlsbad, CA). The construct was further sequenced to verify the orientation and integrity of the ligated *HOXC13-AS* insert.

RNA pulldown

Full-length *HOXC13-AS* was transcribed *in vitro* using the T7 MEGAscript kit (ThermoFisher Scientific) and labeled using the Pierce RNA 3′ End Desthiobiotinylation Kit (ThermoFisher Scientific). *HOXC13-AS* pulldown assay was performed using the Pierce Magnetic RNA-Protein Pull-Down Kit (ThermoFisher Scientific) according to the manufacturer's instructions. Briefly, 50 pmol of desthiobiotinylated *HOXC13-AS* was mixed with magnetic beads and incubated with 200 µg of protein lysate from human adult epidermal keratinocytes (ThermoFisher Scientific). Poly(A)$_{25}$ RNA, which was included in the RNA-Protein Pull-Down kit, was used as a negative control. The beads were washed, and RNA-bound proteins were eluted by boiling the beads in SDS buffer. Eluted protein samples were run on the Mini-PROTEAN TGX gels (Bio-Rad) followed by silver staining. Specific bands were excised and analyzed by mass spectrometry at the Proteomics Biomedicum core facility, Karolinska Institute.

In-gel protein digestion and mass spectrometry

Protein bands were excised manually from gels and in-gel digested using a MassPREP robotic protein-handling system (Waters, Millford, MA). Gel pieces were distained following the manufacturer's description. Proteins then were reduced with 10 mM DTT in 100 mM Ambic for 30 min at 40°C and alkylated with 55 mM iodoacetamide in 100 mM Ambic for 20 min at 40°C followed by digestion with

0.3 μg trypsin (sequence grade, Promega, Madison, WI) in 50 mM Ambic for 5 hours at 40°C. The tryptic peptides were extracted with 1% formic acid in 2% acetonitrile, followed by 50% acetonitrile twice. The liquid was evaporated to dryness on a vacuum concentrator (Eppendorf).

The reconstituted peptides in solvent A (2% acetonitrile, 0.1% formic acid) were separated on a 50 cm long EASY-spray column (ThermoFisher Scientific) connected to an Ultimate-3000 nano-LC system (ThermoFisher Scientific) using a 60 min gradient from 4-26% of solvent B (98% acetonitrile, 0.1% formic acid) in 55 min and up to 95% of solvent B in 5 min at a flow rate of 300 nL/min. Mass spectra were acquired on a Q Exactive HF hybrid Orbitrap mass spectrometer (ThermoFisher Scientific) in m/z 375 to 1500 at resolution of R=120,000 (at m/z 200) for full mass, followed by data-dependent HCD fragmentations from most intense precursor ions with a charge state 2+ to 7+. The tandem mass spectra were acquired with a resolution of R=30,000, targeting $2x10^5$ ions, setting isolation width to m/z 1.4 and normalized collision energy to 28%.

Acquired raw data files were analyzed using the Mascot Server v.2.5.1 (Matrix Science Ltd., UK) and searched against SwissProt protein databases (20,368 human entries). Maximum of two missed cleavage sites were allowed for trypsin, while setting the precursor and the fragment ion mass tolerance to 10 ppm and 0.02 Da, respectively. Dynamic modifications of oxidation on methionine, deamidation of asparagine and glutamine and acetylation of N-termini were set. Initial search results were filtered with 5% FDR using Percolator to recalculate Mascot scores. Protein identifications were accepted if they could be established at greater than 96.0% probability and contained at least 2 identified peptides. Proteins that contained similar peptides and could not be differentiated based on MS/MS analysis alone were grouped to satisfy the principles of parsimony.

RNA immunoprecipitation

RNA immunoprecipitation (RIP) was performed using the Magna RIP RNA-Binding Protein Immunoprecipitation Kit (Millipore, Burlington, MA). Briefly, human primary keratinocytes were lysed in RIP lysis buffer, and then 100 μl of cell lysate was incubated with an anti-COPA antibody (sc-398099, Santa Cruz Biotechnology). Mouse IgG, instead of anti-COPA antibody, was used as a negative control. The immunoprecipitated RNA was extracted and analyzed using RT-PCR followed by electrophoresis or qRT-PCR. SNRNP70 antibody and U1 snRNA primers provided by the kit were used as a positive control.

Statistics

Data are representatives of at least two independent experiments, and the number of biological replicates was shown in figure legends. Data analysis was performed by using GraphPad Prism Version 7 (Dotmatics, San Diego, CA). Statistical significance among the two groups was determined by a two-tailed Student's t-test or Mann-Whitney Test. The significance among multiple groups was determined by one-way ANOVA with Bonferroni posttest. Pearson's correlation test was performed on log2-transformed data. $P < 0.05$ was considered statistically significant.

Schematics

Schematic cartoons in 1a, 2b, 5a, 6b, 6j, 8e, and Supplementary Fig. 3k were created with BioRender.com.

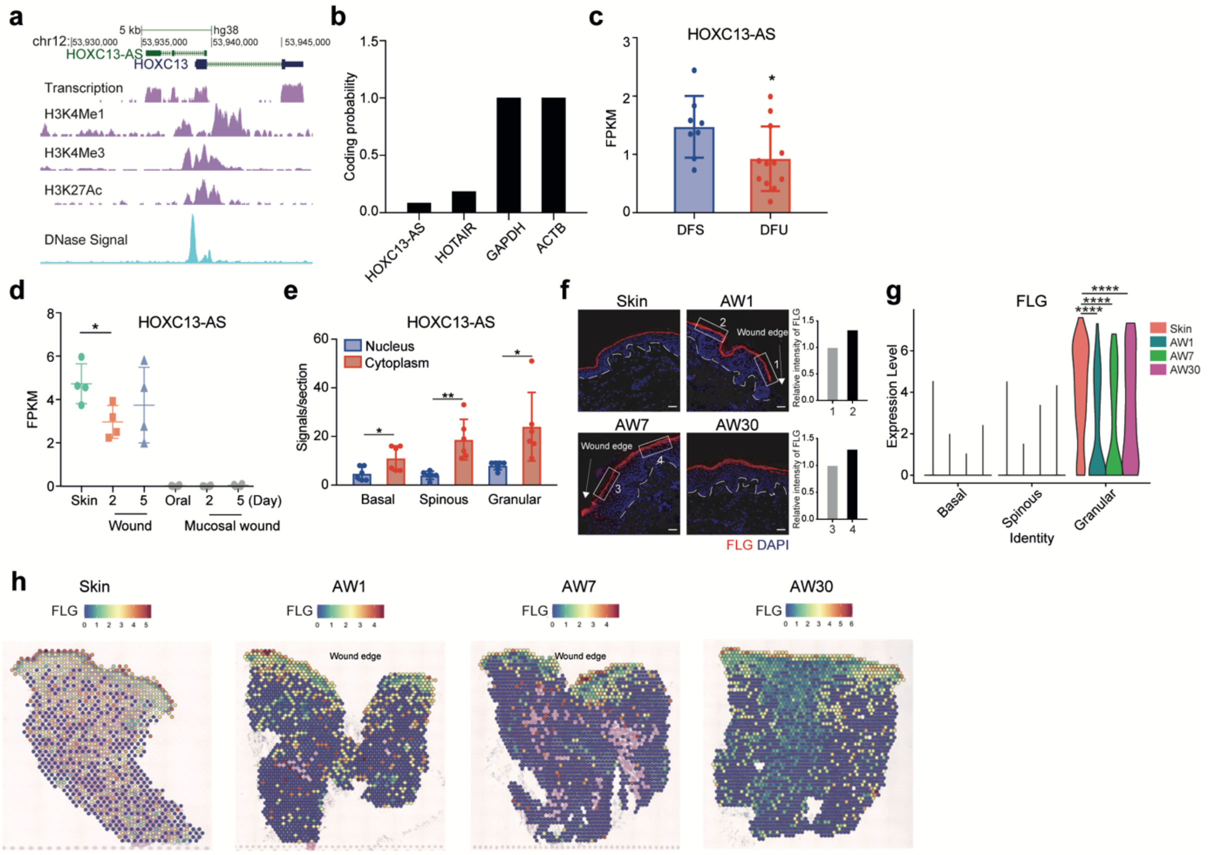

Supplementary Figure 1 Characterization of *HOXC13*-AS and analysis of FLG expression in human skin. a UCSC genome browser tracks depicting transcription, DNase hypersensitivity regions, and histone modifications (H3K4me1, H3K4me3, H3K27ac) in *HOXC13-AS* and *HOXC13* loci. **b** Bar graph representing coding potential scores obtained from Coding Potential Calculator 2 for coding (*GAPDH* and *ACTB*) and non-coding (*HOXC13-AS* and *HOTAIR*) genes. Querying *HOXC13-AS* expression in the published RNA-seq datasets of human wounds: diabetic foot ulcers (DFU) vs. diabetic foot skin (DFS) (GSE134431) **c**; day -2 and day-5 skin wounds vs. day -2 and day-5 mucosal wounds (GSE97615) **d**. Data are normalized as Fragments per kilobase of a transcript, per million mapped reads (FPKM). **e** Quantification of the subcellular localization of *HOXC13-AS* in different layers of human skin epidermis analyzed by FISH (n = 6 sections). **f** Immunofluorescence staining of FLG in human skin and acute wounds. Relative intensity of FLG signals in the rectangles was analyzed. Scale bar = 50 µm. **g** Violin plots of *FLG* expression in keratinocytes at different epidermal layers of human skin and acute wounds analyzed by scRNA-seq. **h** Visualization of *FLG* expression in human skin and acute wounds by spatial transcriptomics. *P < 0.05, **P < 0.01, and ****P < 0.0001 by unpaired two-tailed Student's t test **c**, **d**, **e**, or One-way ANOVA **g**. Data are presented as mean ± SD **c**, **d**, **e**, or violin plots **g**.

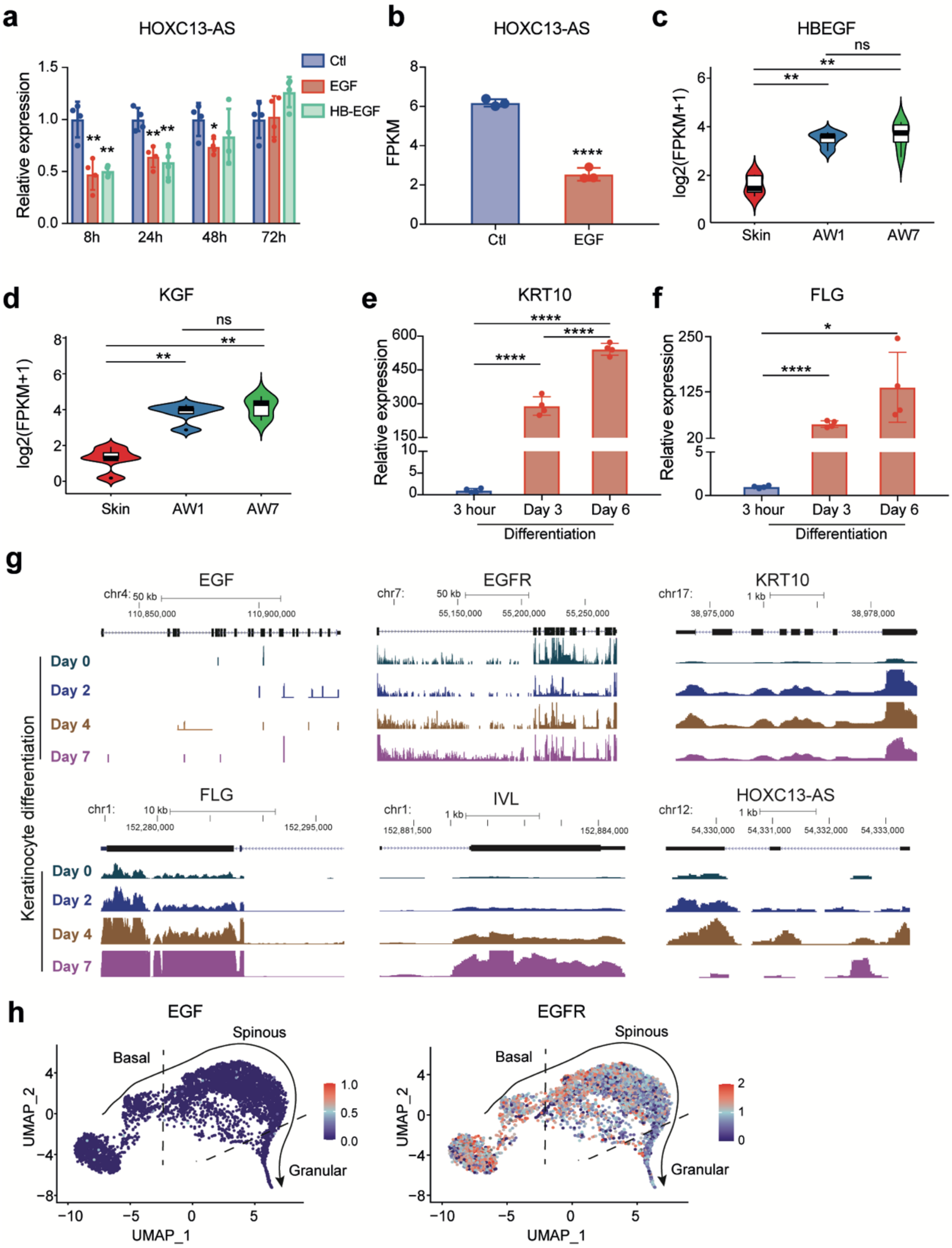

Supplementary Figure 2 *HOXC13-AS* expression in keratinocytes is oppositely regulated by growth and differentiation signals. a QRT-PCR analysis of *HOXC13-AS* expression in human primary keratinocytes treated with EGF or HBEGF for 8-72 hours (n = 4). **b** Querying *HOXC13-AS* expression in the published RNA-seq datasets of epidermal stem cells treated with EGF (GSE156089). RNA-seq analysis of *HBEGF* **c** and *KGF* expression **d** in human skin and day-1 and day-7 acute wounds, retrieved from the public dataset(11). QRT-PCR analysis of *KRT10* **e** and *FLG* **f** expression in calcium-induced differentiated keratinocytes (n = 4). **g** UCSC genome browser view of *EGF*, *EGFR*, *KRT10*, *FLG*, *IVL*, and *HOXC13-AS* in the normalized RNA-seq data for keratinocyte differentiation at multiple time points, retrieved from GEO GSE59827(12). **h** *EGF* and *EGFR* expression in pseudotime trajectory

of keratinocytes in human skin analyzed by single-cell RNA-seq. *$P < 0.05$; **$P < 0.01$ and ****$P < 0.0001$ by unpaired two-tailed Student's t test **a**, **b**, **e**, **f**, or Mann Whitney test **c**, **d**. Data are presented as mean ± SD **a**, **b**, **e**, **f** or violin plots **c**, **d**.

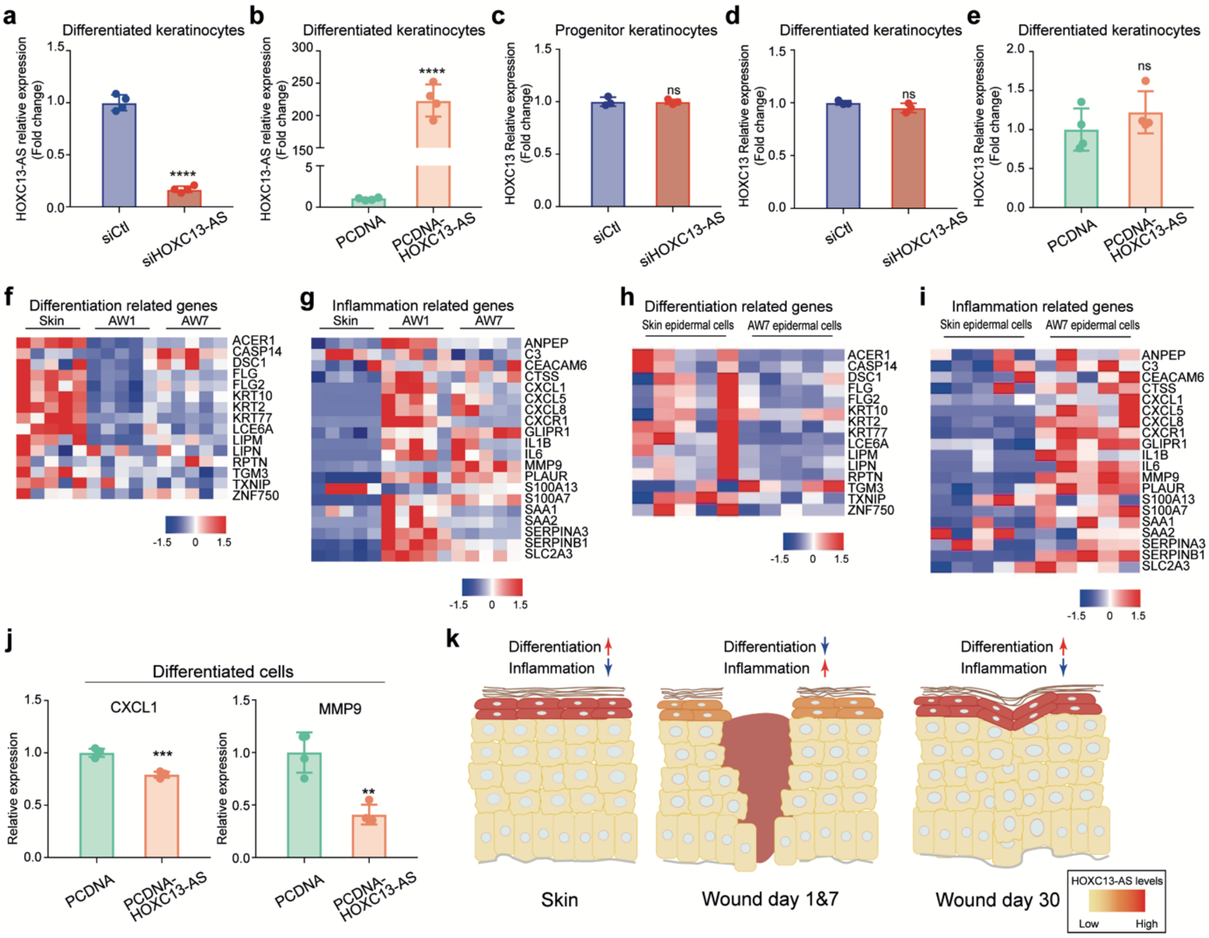

Supplementary Figure 3 *HOXC13-AS* regulates keratinocyte differentiation and inflammatory response. QRT-PCR analysis of *HOXC13-AS* expression in keratinocyte transfected with *HOXC13-AS* siRNA pool **a**, or pcDNA-*HOXC13-AS* **b** (n = 4). QRT-PCR analysis of *HOXC13* expression in keratinocyte transfected with *HOXC13-AS* siRNA pool **c**, **d**, or pcDNA-*HOXC13-AS* **e** in progenitor or differentiated keratinocytes (n = 3-4). Heatmaps showing the expression of *HOXC13-AS* regulated genes in the skin and wound tissue biopsies **f**, **g** and the isolated epidermal cells **h**, **i** analyzed by RNA-seq. **j** QRT-PCR analysis of *CXCL1* and *MMP9* in differentiated keratinocytes with *HOXC13-AS* overexpression (n = 4). **k** Schematic representation of the changes of *HOXC13-AS* expression and its regulated biological processes during human skin wound healing. ns: not significant, $^{**}P < 0.01$, $^{***}P < 0.001$ and $^{****}P < 0.0001$ by unpaired two-tailed Student's t test **a**-**e**, **j**. Data are presented as mean ± SD.

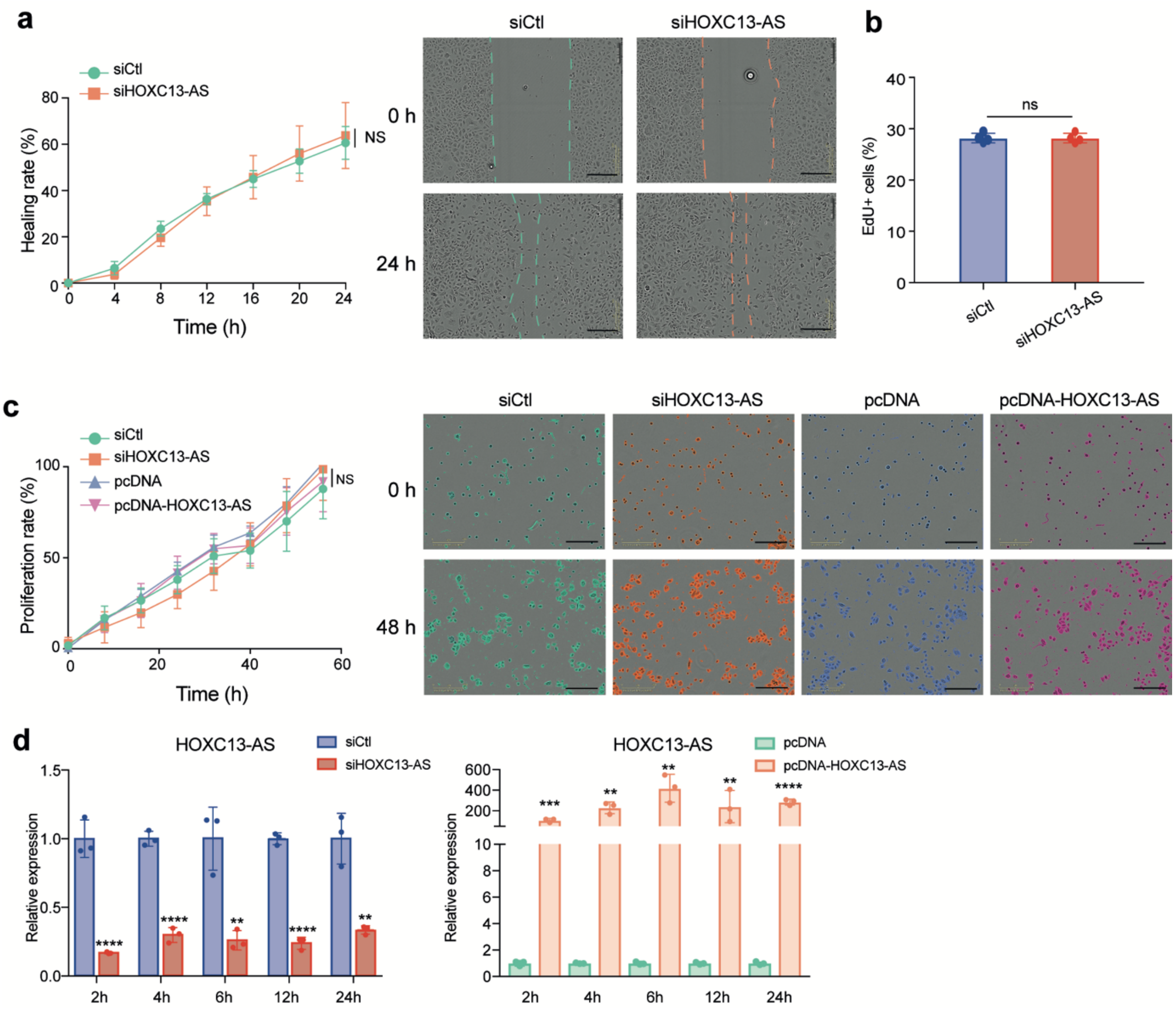

Supplementary Figure 4 ***HOXC13-AS*** **does not affect keratinocyte growth, migration, or apoptosis. a** The migration of keratinocytes with *HOXC13-AS* knockdown (n = 8) was analyzed using a live cell imaging system. Representative photographs of cells at 0 and 24 hours are shown (Scale bar, 300 μm). **b** 5-ethynyl-2'-deoxyuridine (EdU) incorporation assay of keratinocytes with *HOXC13-AS* silencing (n = 4). **c** The growth of keratinocytes with *HOXC13-AS* knockdown or overexpression (n = 3) was analyzed using a live cell imaging system. Representative photographs of cells at 0 and 96 hours are shown (Scale bar, 300 μm.). **d** QRT-PCR analysis of *HOXC13-AS* expression in keratinocytes with *HOXC13-AS* knockdown or overexpression followed by suspension induction for 2-24 hours (n = 3). ns: not significant, ***P* < 0.01, ****P* < 0.001, and *****P* < 0.0001 by unpaired two-tailed Student's t test **b**, **d**, or one-way ANOVA **a**, **c**. Data are presented as mean ± SD.

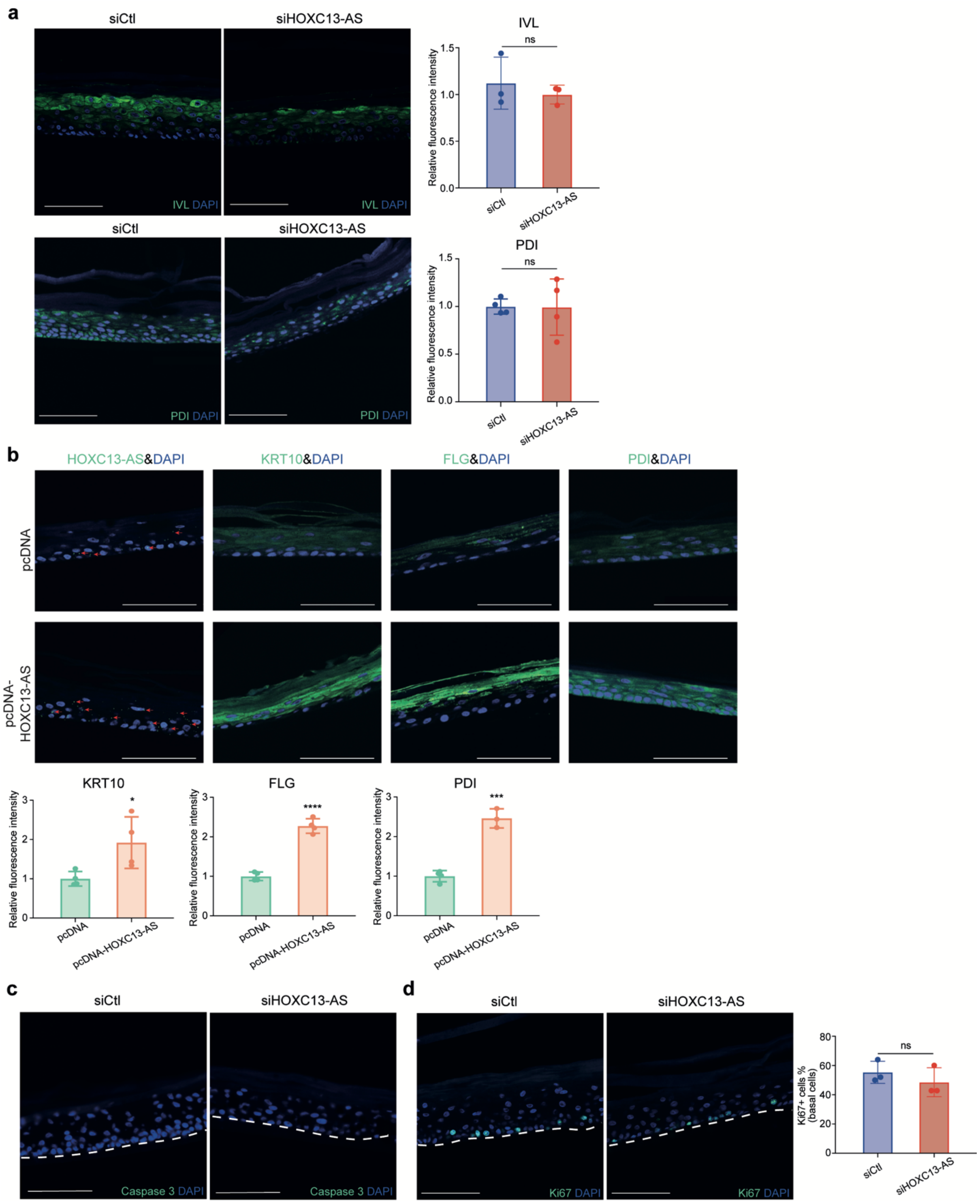

Supplementary Figure 5 *HOXC13-AS* regulates keratinocyte differentiation in organotypic human epidermal tissues. Immunofluorescence staining of IVL and PDI **a**, caspase 3 **c**, and Ki67 **d** in organotypic human epidermal tissues with *HOXC13-AS* KD (scale bar = 100 μm, n = 3-4). **b** Immunofluorescence staining of KRT10, FLG, PDI, and FISH of *HOXC13-AS* in organotypic human epidermal tissues with *HOXC13-AS* OE (scale bar = 100 μm, n = 3-4). ns: not significant, *P < 0.05, ***P < 0.001, and ****P < 0.0001 by unpaired two-tailed Student's t test **a**, **b**, **d**. Data are presented as mean ± SD.

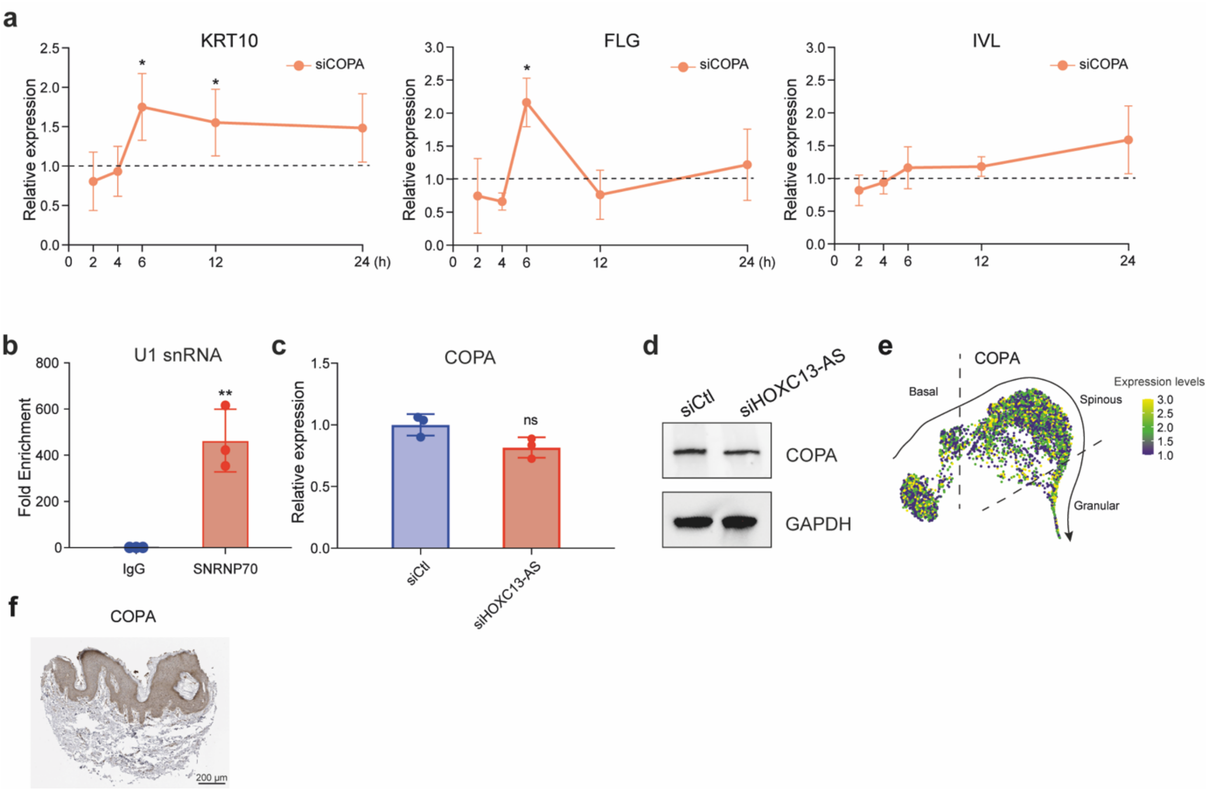

Supplementary Figure 6 The effect of COPA silencing on keratinocyte differentiation and the impact of *HOXC13-AS* on COPA expression. a QRT-PCR analysis of *KRT10*, *FLG*, and *IVL* in keratinocytes transfected with COPA siRNA, followed by suspension-induced differentiation. The results were normalized with the respective controls (n = 3). **b** QRT-PCR analysis of U1 snRNA retrieved from RIP using SNRNP70 antibody (n = 3). QRT-PCR analysis **c** and western blot **d** of COPA levels in keratinocytes transfected with *HOXC13-AS* siRNA pool. **e** *COPA* expression in pseudotime trajectory of the keratinocytes in human skin analyzed by single-cell RNA-seq. **f** Immunohistochemistry staining of COPA in human skin retrieved from the human protein atlas(13) (Human Protein Atlas proteinatlas.org). ns: not significant, **P* < 0.05, and ***P* < 0.01 by unpaired two-tailed Student's t test **a** - **c**. Data are presented as mean ± SD.

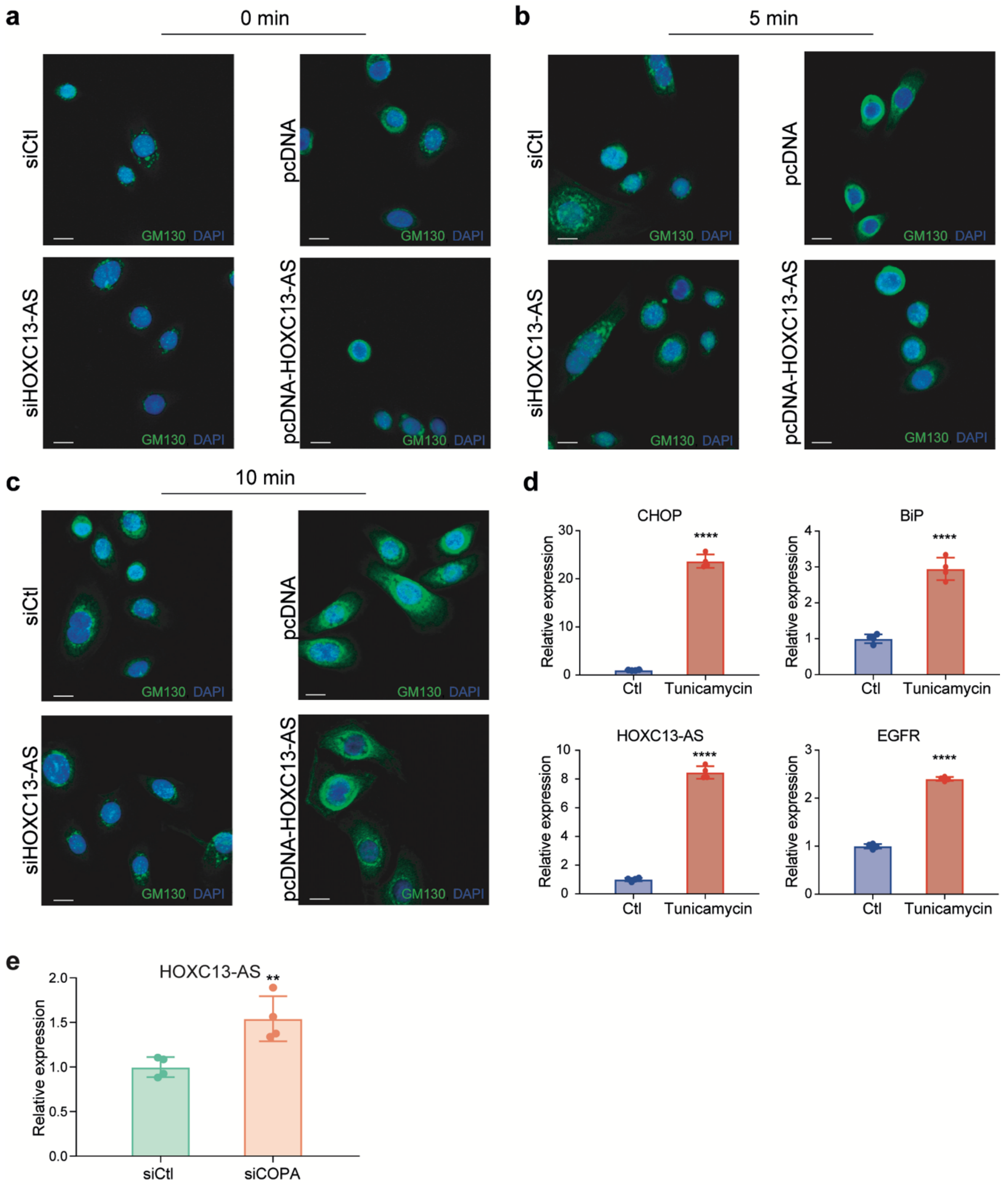

Supplementary Figure 7 *HOXC13-AS* interferes with Golgi-ER retrograde transport and its expression is induced by ER stress. Representative photograph of GM130 immunofluorescence staining in keratinocytes transfected with *HOXC13-AS* siRNA pool, pcDNA-*HOXC13-AS,* or respective controls, and treated with Brefeldin A for 0 minute **a**, 5 minutes **b,** and 10 minutes **c**. Cell nuclei were co-stained with DAPI. Scale bar = 10 μm. **d** QRT-PCR analysis of *CHOP*, *BiP*, *HOXC13-AS*, and *EGFR* in keratinocytes treated with tunicamycin for 24 hours (n = 4). **e** QRT-PCR analysis of *HOXC13-AS* in keratinocytes transfected with COPA siRNA (n = 4). ns: not significant, $^{**}P < 0.01$, and $^{****}P < 0.0001$ by unpaired two-tailed Student's t test **d**, **e**. Data are presented as mean ± SD.

Supplementary Table 1. Donor information

Donor	Sex	Age (years)	Experiments
1	F	66	RNA sequencing
2	M	69	RNA sequencing
3	F	67	RNA sequencing
4	M	69	RNA sequencing, qRT-PCR
5	F	64	RNA sequencing, qRT-PCR
6	F	60	qRT-PCR
7	F	66	qRT-PCR
8	F	60	qRT-PCR
9	F	67	qRT-PCR
10	F	65	qRT-PCR
11	M	26	Cell isolation, RNA sequencing
12	F	30	Cell isolation, RNA sequencing
13	M	45	Cell isolation, RNA sequencing
14	F	43	Cell isolation, RNA sequencing
15	M	22	Cell isolation, RNA sequencing
16	F	48	Cell isolation, qRT-PCR, IF
17	M	27	Cell isolation, qRT-PCR
18	F	24	Cell isolation, qRT-PCR
19	M	24	Cell isolation, qRT-PCR
20	F	46	Cell isolation, qRT-PCR
21	F	24	FISH
22	F	22	FISH, sc-RNA-seq
23	M	29	sc-RNA-seq
24	M	24	sc-RNA-seq
25	F	46	Spatial transcriptomics

M, male; F, female

Supplementary Table 2. List of cytokines, growth factors and chemicals used in the study

Cytokines/Growth factors/Chemicals	Cat.no	Concentration	Vendor
EGF	11343406	20ng/mL	ImmunoTools
FGF-2	11343625	30ng/mL	ImmunoTools
GM-CSF	11343123	50ng/mL	ImmunoTools
HB-EGF	259-HE-050	20ng/mL	ImmunoTools
IGF-1	11343314	20ng/mL	ImmunoTools
IL-36α	11340362	10ng/mL	ImmunoTools
IL-6	11340060	50ng/mL	ImmunoTools
IL1α	11349013	20ng/mL	ImmunoTools
IL23	11340232	10ng/mL	ImmunoTools
KGF	11343653	20ng/mL	R&D Systems
MCP-1	11343380	10ng/mL	ImmunoTools
TGFB1	11343161	10ng/mL	ImmunoTools
TGFB3	11344482	10ng/mL	ImmunoTools
TNF-α	210-TA	50ng/mL	R&D Systems
VEGF	11343663	20ng/mL	ImmunoTools
Tunicamycin	T7765	2µM	MERCK
PERK inhibitor	516535	250nM	MERCK
ISR Inhibitor	5095840001	200nM	MERCK

Supplementary Table 3. **List of siRNA pools used in the study**

siRNAs	Cat.no/Vendor
siHOXC13-AS pool	R-188045-00-0005, Dharmacon™, Lafayette, CO
Lincode Non-targeting pool	D-001320-10-05, Dharmacon™
Non-Targeting pool	D-001810-10-05, Dharmacon™
EIF3A	L-0195300-0005, Dharmacon™
HDLBP	L-019956-00-0005, Dharmacon™
COPA	L-011835-00-0005, Dharmacon™

Supplementary Table 4. **List of primers used in the study**

Gene	Sequence/Cat.no/Vendor
HOXC13-AS	F 5' CGGAGAAAGACGGAGCATTTA<br>R 5' CAGAGTTGAACTTAGCCGAGAG, IDT
KRT10	Hs.PT.58.38635764, IDT
FLG	F 5' ATGAGCAGGCACGAAACA<br>R 5' CCTGAGTGTCCAGACCTATCTA, IDT
IVL	Hs.PT.58.39460547, IDT
MALAT1	F 5' GATCTAGCACAGACCCTTCAC<br>R 5'CGACACCATCGTTACCTTGA, IDT
sXBP1	356738665 Rxn Ready Primer Pool, IDT
tXBP1	356738667 Rxn Ready Primer Pool, IDT
ATF4	356738668 Rxn Ready Primer Pool, IDT
CHOP	356738669 Rxn Ready Primer Pool, IDT
BiP	356738670 Rxn Ready Primer Pool, IDT
EDEM	356738672 Rxn Ready Primer Pool, IDT
IL6	Hs.PT.5840226675, IDT
IL8	Hs.PT.5839926886.g, IDT
IL1B	Hs.PT.581518186, IDT
B2M	F 5' AAGTGGGATCGAGACATGTAAG<br>R 5' GGAGACAGCACTCAAAGTAGAA, IDT
GAPDH	HS.PT.39A.22214836(1), IDT<br>F 5' GGTGTGAACCATGAGAAGTATGA<br>R 5' GAGTCCTTCCACGATACCAAAG, IDT

Reference

1. Hao Y, Hao S, Andersen-Nissen E, Mauck WM, 3rd, Zheng S, Butler A, et al. Integrated analysis of multimodal single-cell data. Cell. 2021;184(13):3573-87 e29.
2. Hafemeister C, Satija R. Normalization and variance stabilization of single-cell RNA-seq data using regularized negative binomial regression. Genome Biol. 2019;20(1):296.
3. Korsunsky I, Millard N, Fan J, Slowikowski K, Zhang F, Wei K, et al. Fast, sensitive and accurate integration of single-cell data with Harmony. Nat Methods. 2019;16(12):1289-96.
4. Bolger AM, Lohse M, Usadel B. Trimmomatic: a flexible trimmer for Illumina sequence data. Bioinformatics. 2014;30(15):2114-20.
5. Dobin A, Davis CA, Schlesinger F, Drenkow J, Zaleski C, Jha S, et al. STAR: ultrafast universal RNA-seq aligner. Bioinformatics. 2013;29(1):15-21.
6. Liao Y, Smyth GK, Shi W. featureCounts: an efficient general purpose program for assigning sequence reads to genomic features. Bioinformatics. 2014;30(7):923-30.
7. Love MI, Huber W, Anders S. Moderated estimation of fold change and dispersion for RNA-seq data with DESeq2. Genome Biol. 2014;15(12):550.
8. Vietri Rudan M, Mishra A, Klose C, Eggert US, Watt FM. Human epidermal stem cell differentiation is modulated by specific lipid subspecies. Proc Natl Acad Sci U S A. 2020;117(36):22173-82.
9. Subramanian A, Tamayo P, Mootha VK, Mukherjee S, Ebert BL, Gillette MA, et al. Gene set enrichment analysis: a knowledge-based approach for interpreting genome-wide expression profiles. Proc Natl Acad Sci U S A. 2005;102(43):15545-50.
10. Panatta E, Lena AM, Mancini M, Smirnov A, Marini A, Delli Ponti R, et al. Long non-coding RNA uc.291 controls epithelial differentiation by interfering with the ACTL6A/BAF complex. EMBO Rep. 2020;21(3):e46734.
11. Liu Z, Zhang L, Toma MA, Li D, Bian X, Pastar I, et al. Integrative small and long RNA omics analysis of human healing and nonhealing wounds discovers cooperating microRNAs as therapeutic targets. eLife. 2022;11:e80322.
12. Kouwenhoven EN, Oti M, Niehues H, van Heeringen SJ, Schalkwijk J, Stunnenberg HG, et al. Transcription factor p63 bookmarks and regulates dynamic enhancers during epidermal differentiation. EMBO Rep. 2015;16(7):863-78.
13. Uhlen M, Fagerberg L, Hallstrom BM, Lindskog C, Oksvold P, Mardinoglu A, et al. Proteomics. Tissue-based map of the human proteome. Science. 2015;347(6220):1260419.
