# Supplementary material for: Human skin specific long noncoding RNA HOXC13-AS regulates epidermal differentiation by interfering with Golgi-ER retrograde transport
Source: Cell Death Differ. 2023 Mar 3;30(5):1334–48. doi: 10.1038/s41418-023-01142-z (PMC10154349; doi:10.1038/s41418-023-01142-z)

Western blot related to Fig. 5i

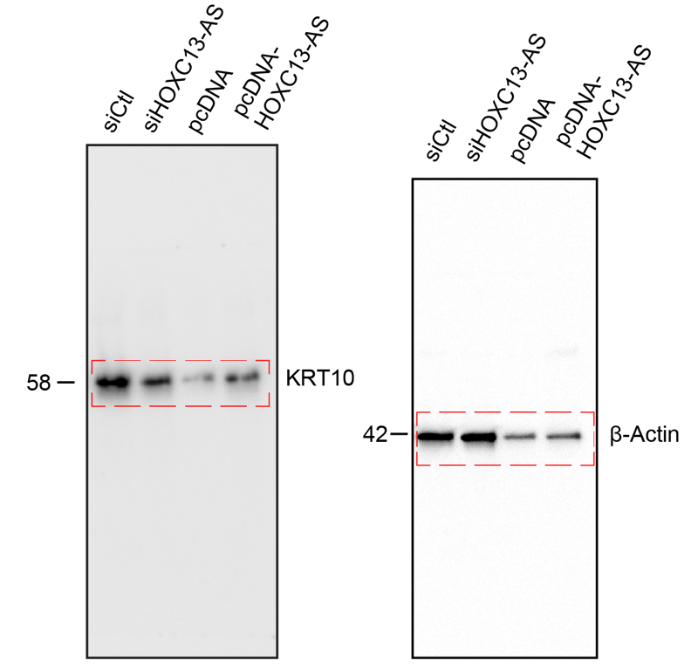

Western blot related to Fig. 6g

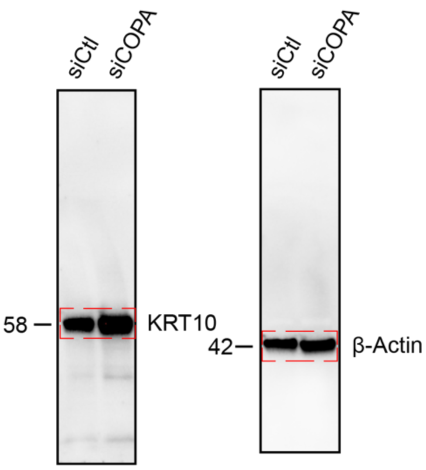

Western blot related to Fig. 6i

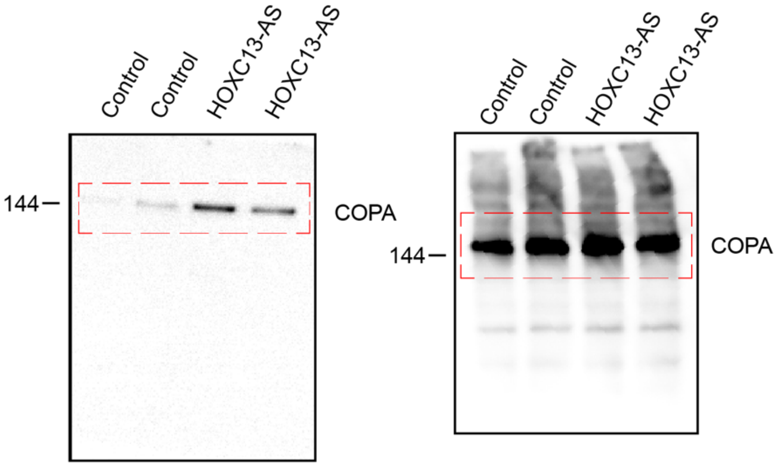

Western blot related to Supplementary Fig. 6d

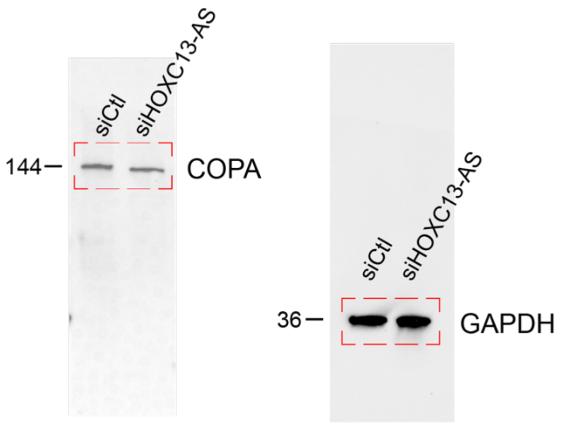

Western blot related to Fig. 7h

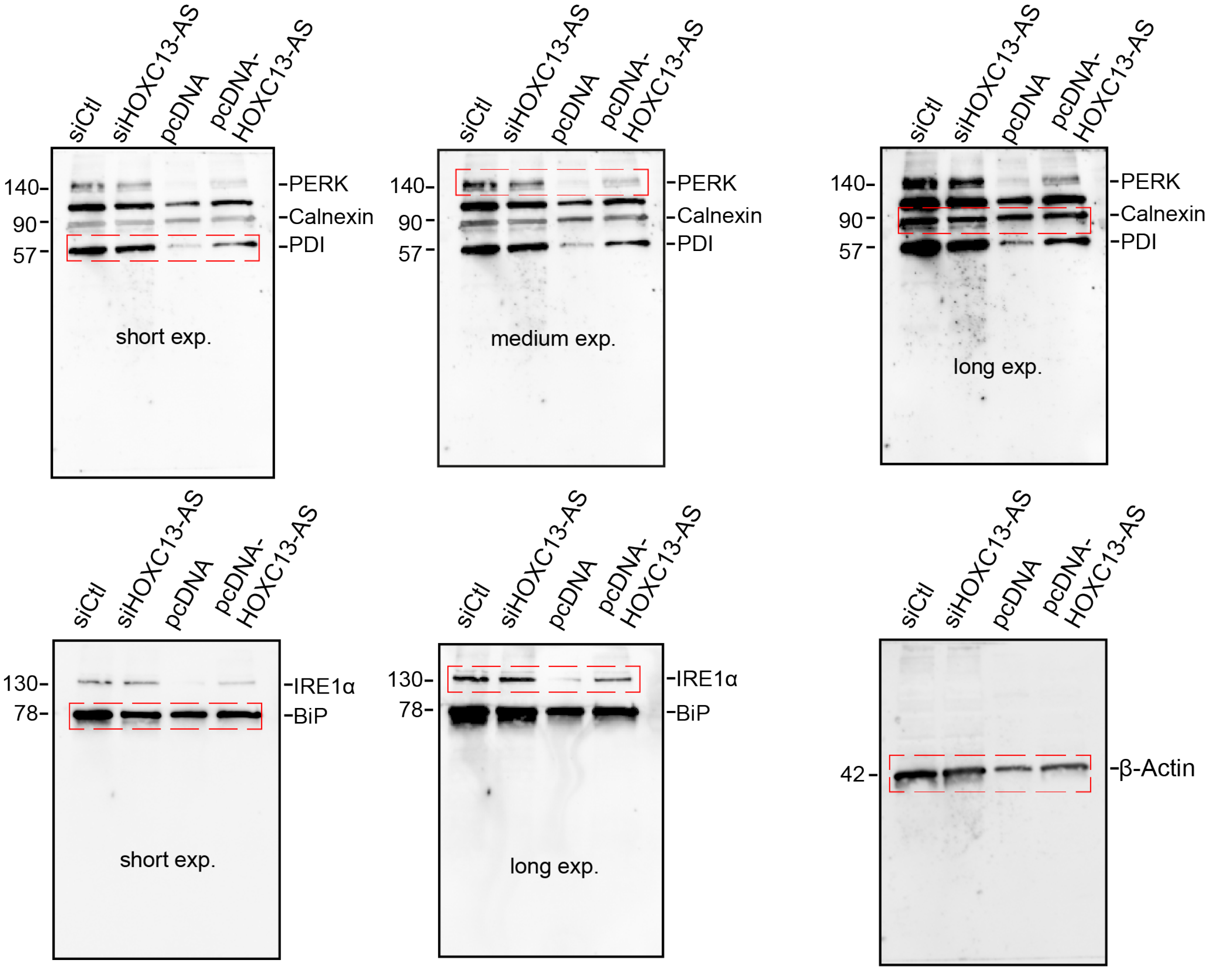

Western blot related to Fig. 7j

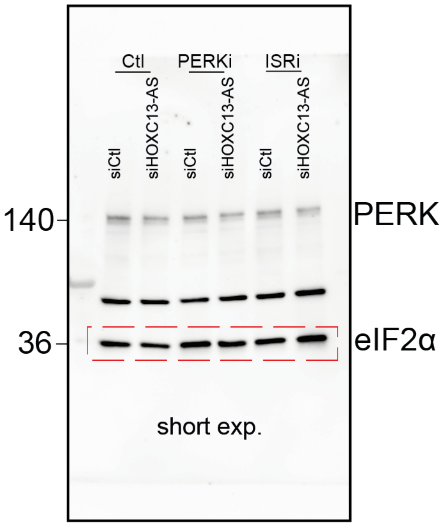

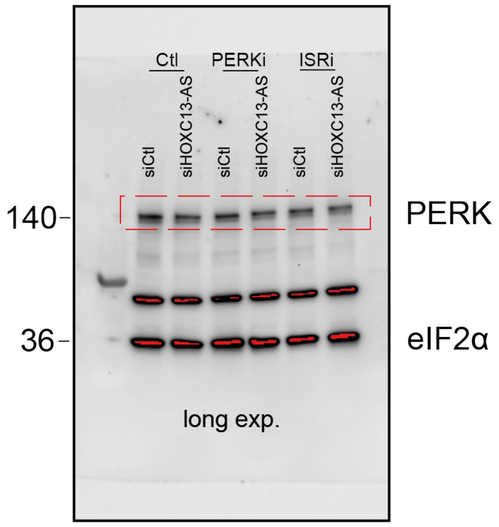

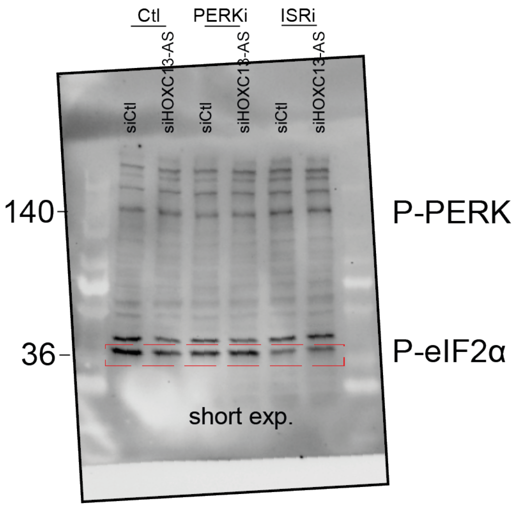

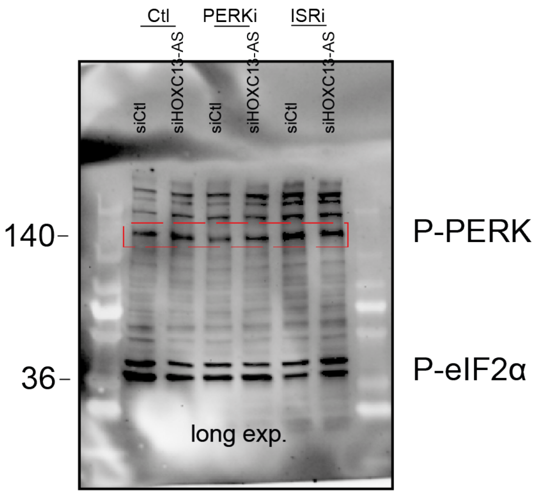

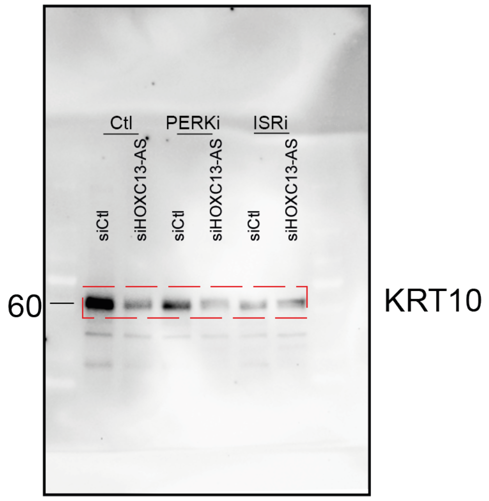

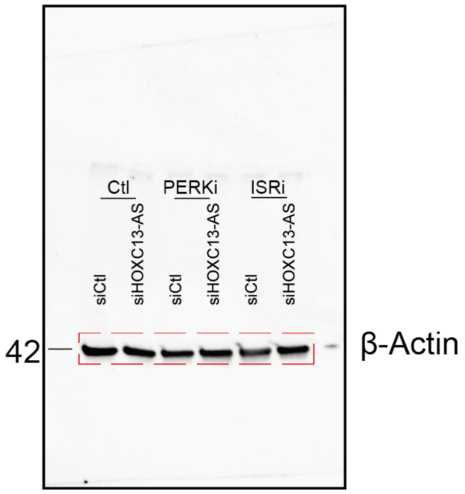

Supplement: Supplementary file 3 — Uncropped WB [file 41418_2023_1142_MOESM3_ESM.pdf]
